# *Caenorhabditis elegans* HIM-18/SLX-4 Interacts with SLX-1 and XPF-1 and Maintains Genomic Integrity in the Germline by Processing Recombination Intermediates

**DOI:** 10.1371/journal.pgen.1000735

**Published:** 2009-11-20

**Authors:** Takamune T. Saito, Jillian L. Youds, Simon J. Boulton, Monica P. Colaiácovo

**Affiliations:** 1 Department of Genetics, Harvard Medical School, Boston, Massachusetts, United States of America; 2 DNA Damage Response Laboratory, Cancer Research UK, South Mimms, United Kingdom; National Cancer Institute, United States of America

## Abstract

Homologous recombination (HR) is essential for the repair of blocked or collapsed replication forks and for the production of crossovers between homologs that promote accurate meiotic chromosome segregation. Here, we identify HIM-18, an ortholog of MUS312/Slx4, as a critical player required *in vivo* for processing late HR intermediates in *Caenorhabditis elegans*. DNA damage sensitivity and an accumulation of HR intermediates (RAD-51 foci) during premeiotic entry suggest that HIM-18 is required for HR–mediated repair at stalled replication forks. A reduction in crossover recombination frequencies—accompanied by an increase in HR intermediates during meiosis, germ cell apoptosis, unstable bivalent attachments, and subsequent chromosome nondisjunction—support a role for HIM-18 in converting HR intermediates into crossover products. Such a role is suggested by physical interaction of HIM-18 with the nucleases SLX-1 and XPF-1 and by the synthetic lethality of *him-18* with *him-6*, the *C. elegans BLM* homolog. We propose that HIM-18 facilitates processing of HR intermediates resulting from replication fork collapse and programmed meiotic DSBs in the *C. elegans* germline.

## Author Summary

Homologous recombination (HR) is a process that provides for the accurate and efficient repair of DNA double-strand breaks (DSBs) incurred by cells, thereby maintaining genomic integrity. Proper processing of HR intermediates is critical for biological processes ranging from replication fork restart to the accurate partitioning of chromosomes during meiotic cell divisions. This is further emphasized by the fact that impaired processing of HR intermediates in both mitotic and meiotic cells can result in tumorigenesis and congenital defects. Therefore, the identification of components involved in HR is essential to understand the molecular mechanism of HR. Here, we identify HIM-18/SLX-4 in *C. elegans*, a protein conserved from yeast to humans that interacts with the nucleases SLX-1 and XPF-1 and is required for DSB repair in the germline. Impaired HIM-18 function results in increased DNA damage sensitivity, the accumulation of recombination intermediates, decreased meiotic crossover frequencies, altered late meiotic chromosome remodeling, the formation of fragile connections between homologs, and an increased chromosome nondisjunction. Finally, HIM-18 is localized to both mitotic and meiotic nuclei in wild-type germlines. We propose that HIM-18 function is required during the processing of late HR intermediates resulting from replication fork collapse and meiotic DSBs.

## Introduction

DNA double-strand breaks (DSBs) can arise in various ways, including as a result of the collapse of stalled replication forks, exposure to DNA damaging agents and the formation of programmed meiotic DSBs [1]. The importance of DSB repair is therefore highlighted by its critical roles in replication fork restart, the maintenance of genomic integrity and promoting faithful meiotic chromosome segregation. Homologous recombination (HR) provides an efficient and accurate repair of DSBs, in part through the use of an intact donor template for repair. Current models of HR propose that following DSB formation, the DSB ends are resected in a 5′ to 3′ orientation generating 3′ single-stranded DNA (ssDNA) tails [2],[3]. Repair can then proceed through different pathways, one of which involves the association of the Rad51 recombinase protein with the ssDNA tails. This generates a nucleoprotein filament that engages in strand invasion with either an intact sister or homologous chromosome resulting in the formation of a D-loop structure. Subsequent second end capture, DNA synthesis and ligation, results in the formation of two four-way DNA junction intermediates referred to as Holliday junctions (HJ). Double HJ (dHJ) intermediates can be resolved through cleavage by HJ resolvases, which results in either a crossover (CO) or noncrossover (NCO) product, or undergo dissolution mediated by RecQ helicases in combination with topoisomerase activity, resulting in NCOs.

Identifying components involved in the processing of HR intermediates has been critical to understand the molecular mechanisms of HR. HJ resolvases have been identified in poxviruses (A22R), bacteriophages (T4 endonuclease VII and T7 endonuclease I), and *E. coli* (RuvC) [4]. In *S. cerevisiae* and *S. pombe*, Cce1 and Cce1/Ydc2, respectively, were identified as HJ resolvases that act in the mitochondria [5],[6]. Recently, *H. sapiens* GEN1 and its homolog in *S. cerevisiae*, Yen1, have been reported to resolve HJs *in vitro* via a canonical RuvC-like symmetrical cleavage that does not require further processing [7]. However, their *in vivo* function remains unclear. Moreover, HJ processing can also be achieved through asymmetric cleavage, as exemplified by the biochemical activity of the MUS81-EME1 complex in eukaryotic cells [8],[9].

The identification of proteins involved in HJ processing has been challenging due to the crossfunctionality observed for nucleases throughout different HR pathways. This crossfunctionality can be partly due to changes in complex composition that may modulate specificity of recognition and processing of a given intermediate. In *D. melanogaster*, where 90–95% of meiotic COs do not require MUS81 [10], COs are dependent on a protein complex consisting of MEI-9, an ortholog of the mammalian XPF and *S. cerevisiae* Rad1 nucleotide excision repair (NER) endonucleases, ERCC1, an XPF interaction partner, MUS312 and HDM, a member of a superfamily of proteins with ssDNA binding activity [11]–[16]. However, ERCC1 and HDM are only required for a subset of CO events [12],[13]. In *S. cerevisiae*, *SLX4* was first identified in a synthetic-lethal screen for genes required for viability in the absence of *SGS1*, a member of the RecQ family of DNA helicases implicated in the human Bloom and Werner syndromes [17]. Slx4 binds to two structure-specific endonucleases, Slx1 and Rad1, in a mutually exclusive manner [17],[18]. The Slx1-Slx4 complex cleaves multiple branched DNA substrates *in vitro*, particularly 5′-flap, simple-Y, HJ and replication fork structures [19], and functions in rDNA maintenance during S phase [20],[21]. Meanwhile, the Slx4-Rad1 complex is required for the single-strand annealing (SSA) HR pathway, in which Mec1/Tel1 dependent phosphorylation of Slx4 is essential [18],[22]. Furthermore, an *in vivo* nuclease activity of Slx4 without Slx1 or Rad1 is proposed [19],[23],[24].

Here, through a functional genomics approach in the nematode *C. elegans*, we identified HIM-18, which shares sequence similarity with *S. cerevisiae* Slx4 and *D. melanogaster* MUS312. HIM-18 is localized to mitotic nuclei at the premeiotic tip and to meiotic nuclei from late pachytene through diakinesis in wild type germlines. The accumulation of HR intermediates stemming from replication fork collapse in mitosis and from SPO-11-dependent programmed meiotic DSBs in mid to late pachytene observed in *him-18* mutant germlines, coupled with the localization pattern of HIM-18, suggests a role in both mitotic and meiotic DSB repair. This is further supported by the observation of reduced CO frequencies and the premature disassembly of bivalent attachments at prometaphase I resulting in increased chromosome nondisjunction in *him-18* mutants. Moreover, HIM-18 interacts with SLX-1 and XPF-1, and *him-18* mutants show similar DNA damage sensitivity and synthetic lethality with *him-6/BLM* as observed in *slx4* mutants in yeast. Taken together, our analysis suggests that HIM-18 is required for the maintenance of genomic integrity in germline nuclei. We propose a model in which HIM-18/SLX-4 promotes the processing of late HR intermediates in the germline resulting from replication fork collapse in mitotic nuclei and programmed DSBs in meiotic nuclei.

## Results

### HIM-18 Is a Conserved Chromosome-Associated Protein Present in Both Mitotic and Late Meiotic Prophase Germline Nuclei

**Figure 1:**
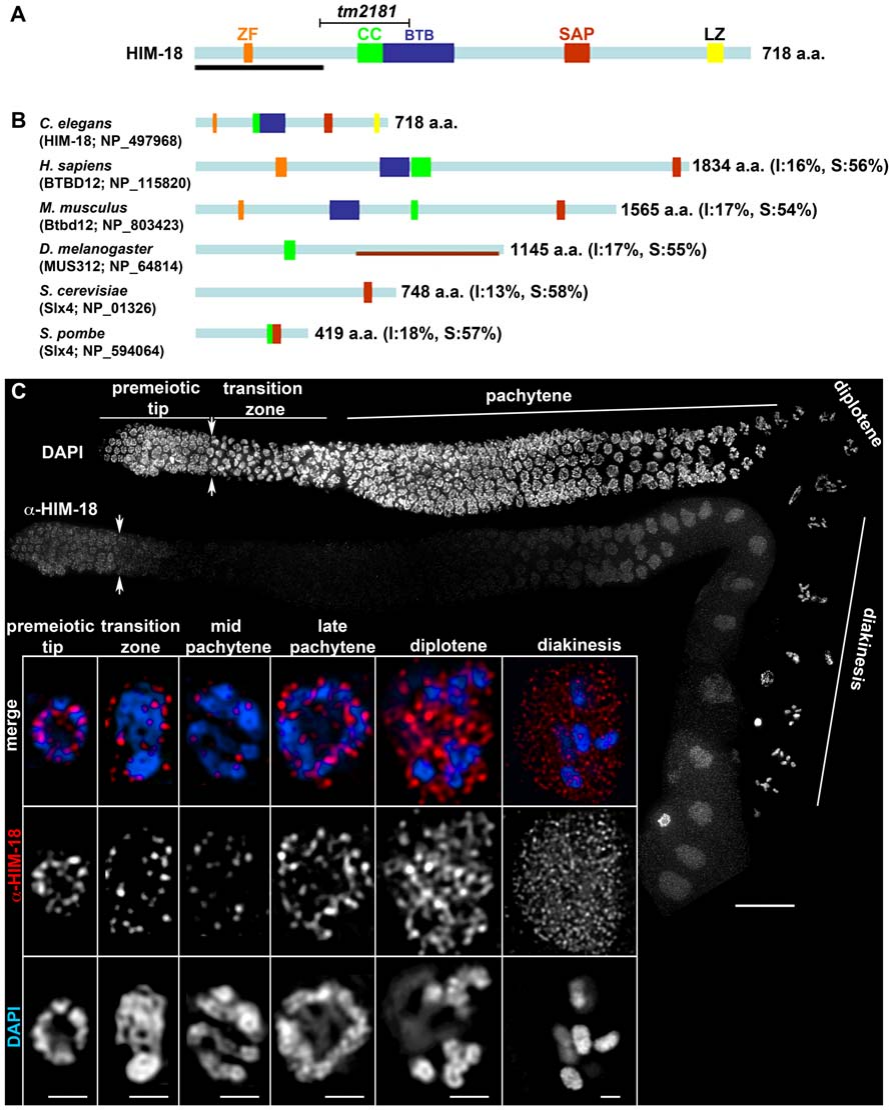
HIM-18 is a conserved protein that localizes in both mitotic and late meiotic prophase germline nuclei. (A) Schematic representation of the predicted HIM-18 protein structure. The region deleted in the *tm2181* mutant allele is indicated (codons 161 through 277). The various conserved domains are indicated by the following abbreviations: ZF, zinc finger; CC, coiled coil; BTB, Bric-a-brac, Tramtrack, Broad-complex; SAP, SAF-A/B, Acinus and PIAS; LZ, leucine zipper. The black bar indicates the N-terminal region used for antibody production. (B) Schematic representation of *Caenorhabditis elegans* HIM-18 and related predicted proteins in *Homo sapiens*, *Mus musculus*, *Drosophila melanogaster*, *Saccharomyces cerevisiae*, and *Schizosaccharomyces pombe.* Protein names and accession numbers are indicated. Percent identity and similarity (I and S, respectively) are indicated in parentheses. The brown horizontal bar indicates the region of homology in Dm-MUS312 shared with Sc-Slx4. (C) Immunolocalization of HIM-18 in germline nuclei of wild type hermaphrodites. Low magnification images show nuclei progressing from the premeiotic tip through all the stages of meiotic prophase, in a left to right orientation. Arrowheads indicate the beginning of transition zone and entrance into meiosis. A single focal plane from a representative nucleus of each stage is shown at a higher magnification. Detection thresholds for α-HIM-18 signals in transition zone and mid-pachytene panels were lower than for other panels to permit imaging of the lower levels of chromosome-associated HIM-18 observed in these stages. Bars, 20 µm for whole gonad, 1 µm in insets.

*him-18* (open reading frame T04A8.15) was identified in an RNA interference (RNAi) screen performed as in [25] and designed to detect meiotic candidates from among germline-enriched genes [[26] and M. Colaiácovo, unpublished results]. The HIM-18 protein harbors two DNA binding motifs (zinc finger and SAP) and three protein binding motifs (coiled-coil, BTB and leucine-zipper) (Figure 1A). Homology searches revealed HIM-18 orthologs from yeast to humans (Figure 1B). Specifically, the SAP motif is highly conserved throughout these orthologs, with the exception of MUS312 in *D. melanogaster*. This conservation is particularly interesting because the SAP motif, present in the *S. pombe* mitochondrial HJ resolvase Cce1/Ydc2, is implicated in promoting HJ binding and resolution [27],[28]. Furthermore, the mammalian orthologs, named BTBD12, also contain BTB and zinc finger motifs. Therefore, this sequence analysis suggests that HIM-18 is conserved among eukaryotic organisms and shares homology with proteins predicted to function in the processing of HR intermediates.

The *him-18*(*tm2181*) mutant, obtained from the Japanese National Bioresource Project, carries a 394 bp out-of-frame deletion encompassing parts of exons 5 and 6 (Figure 1A). This deletion results in a premature stop codon and the loss of the predicted coiled-coil, BTB, SAP and leucine-zipper motifs. The analysis of DAPI-stained germlines of *him-18(tm2181)/+* hermaphrodites, and a genetic analysis of both embryonic lethality and the incidence of males among the progeny of these worms, indicated that these heterozygotes were indistinguishable from wild type (Table 1). This indicates that *tm2181* is a recessive allele of *him-18*. A similar analysis indicated that *tm2181* homozygotes were indistinguishable from transheterozygotes for *tm2181* and *sDf121*, a deficiency encompassing the *him-18* locus (Table 1). Moreover, an affinity purified HIM-18 antibody raised against the first 166 amino acids present in HIM-18, failed to detect a HIM-18 signal on immunostained whole mounted gonads from *him-18(tm2181)* mutants (Figure S1). Taken together, these studies suggest that *him-18(tm2181)* is a null.

**Table 1:** Plate phenotypes.

Genotype	Mean no. of eggs/brood (n)^a^	% Inviable embryos (n)^b^	% Males (n)^c^	% Larval arrest (n)^d^
wild type	297 (12)	0.4 (3567)	0.2 (3553)	0 (3554)
control (RNAi)	300 (12)	0.3 (3595)	0.1 (3575)	0.2 (3583)
*him-18*	196 (14)	79.9 (2748)	11.9 (243)	56.1 (553)
*him-18(RNAi)*	145 (3)	72.5 (436)	5.5 (73)	39.2 (120)
*him-18/+*	312 (11)	0.2 (3437)	0.2 (3413)	0.5 (3431)
*unc-32/+*	310 (10)	2.1 (3095)	0.2 (2998)	1.1 (3031)
*+/sDf121 unc-32*	245 (14)	26.2 (3423)	0.2 (2516)	0.4 (2525)
*him-18/sDf121 unc-32*	188 (10)	82.2 (1881)	17.3 (98)	70.7 (335)
*xpf-1*	235 (10)	20.2 (2348)	4.5 (1718)	8.3 (1873)
*xpf-1(RNAi)*	263 (11)	7.8 (2890)	3.5 (2625)	1.5 (2665)
*xpf-1; him-18*	120 (29)	84.3 (3482)	13.0 (123)	77.4 (545)
*him-6*	235 (9)	59.1 (2270)	13.7 (878)	5.4 (928)
*mus-81*	140 (10)	20.7 (1403)	0.2 (1027)	7.6 (1112)
*mus-81; him-18*	147 (13)	87.8 (1915)	5.6 (54)	76.9 (234)
*him-18; him-6*	10 (30)	100 (313)	ND	ND
*spo-11*	194 (8)	99.5 (1554)	ND	100 (7)
*spo-11; him-18*	203 (13)	99.2 (2633)	ND	100 (20)
*msh-5*	148 (8)	85.6 (1187)	34.8 (66)	61.4 (171)
*him-18; msh-5*	148 (7)	97 (1039)	27.3 (11)	64.5 (31)

Parentheses indicate the total number of:

a singled hermaphrodites for which entire brood sizes were scored,

b fertilized eggs scored,

c adults scored,

d L1-L4 worms.

ND, not determined due to n = 0.

To investigate the localization of HIM-18 in the germline, dissected gonads from wild type hermaphrodites were immunostained with the affinity purified HIM-18 N-terminal antibody (Figure 1C). HIM-18 is first observed in mitotic nuclei at the distal tip region of the germline (premeiotic tip). However, the HIM-18 signal is abruptly reduced as nuclei enter into meiotic prophase, being barely visible in the transition zone (leptotene/zygotene) or in early to mid pachytene nuclei. HIM-18 signal is then clearly detected once again in late pachytene nuclei, persisting through the end of diakinesis. At a higher resolution, the HIM-18 signal is observed as nuclear foci (mainly around the chromosomes) in the premeiotic tip, late pachytene and diplotene/diakinesis stages (Figure 1C). Although the HIM-18 signal is reduced from transition zone through mid-pachytene, a low level of HIM-18 foci, both on and around chromosomes, is still apparent at these stages upon longer exposure (Figure 1C). Finally, HIM-18 is no longer observed localizing onto chromosomes after diakinesis (data not shown).

### Homologous Pairing, Axis Morphogenesis, and Synapsis Are Normal in *him-18* Mutants

**Figure 2:**
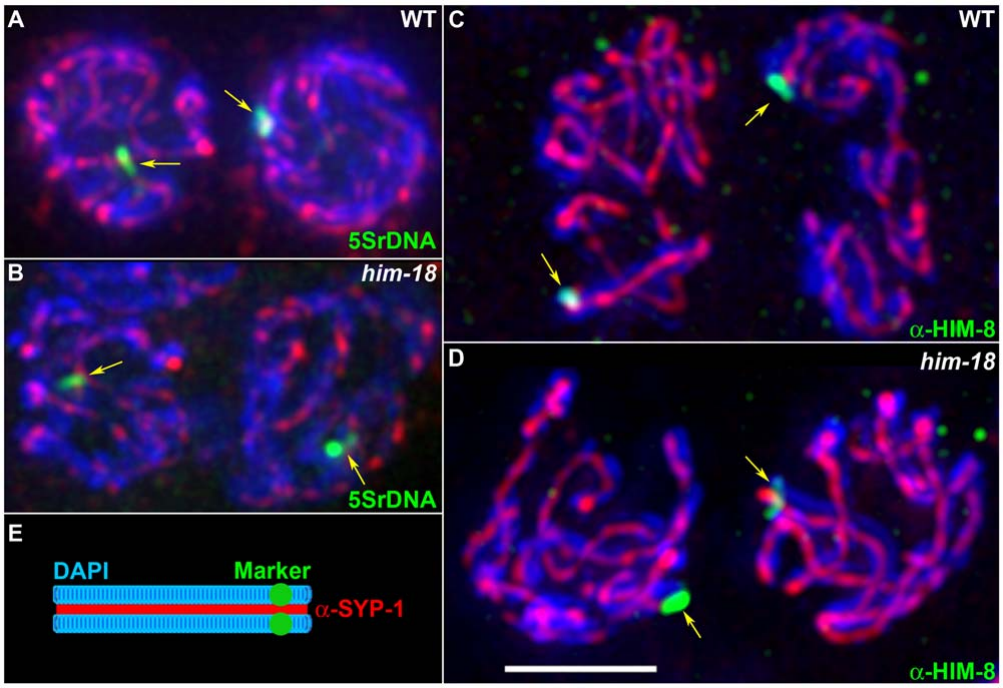
HIM-18 is dispensable for homologous pairing and synapsis. (A, B) Homologous pairing and synapsis were assayed by FISH and immunostaining on squashed mid-pachytene nuclei from wild type and *him-18* mutants. The synaptonemal complex was visualized with the α−SYP-1 antibody and pairing was assessed with a FISH probe targeting the 5S rDNA locus on chromosome V. SYP-1 localizes at the interface between aligned DAPI-stained chromosome pairs. The single or closely juxtaposed hybridization signals observed on each nucleus (yellow arrows) indicate homologous pairing. (C, D) Co-immunostaining with α-SYP-1 and α-HIM-8 to assess synapsis and pairing at the pairing center end of chromosome X in mid-pachytene nuclei. Arrows indicate the single HIM-8 focus observed on each nucleus. (E) Illustration depicting homologous pairing and synapsis. Bar, 2 µm.

Analysis of *him-18(tm2181)* homozygous mutants and following depletion of *him-18* by RNAi revealed phenotypes suggestive of errors in meiotic chromosome segregation such as an increased embryonic lethality (79.9%; n = 2748, and 72.5%; n = 436, respectively) and a high incidence of males (11.9% and 5.5%, respectively) among their surviving progeny (Table 1 and Figure S2A). Errors in meiotic chromosome segregation can stem from earlier defects in homologous chromosome pairing, axis morphogenesis or synapsis. We examined homologous pairing via fluorescence in situ hybridization (FISH) and immunofluorescence analysis, by monitoring both the establishment and maintenance of pairing, as chromosomes enter into meiotic prophase at the transition zone and progress into pachytene where they are fully synapsed. Specifically, we utilized a FISH probe to the 5S ribosomal DNA region (chromosome V) and observed wild type levels of pairing between homologs in *him-18* mutants both in the transition zone and pachytene nuclei (Figure 2A, 2B, and 2E and Figure S3). 98% (n = 107) of the pachytene nuclei examined in *him-18* mutants carried fully paired homologs in this analysis, compared to 97% (n = 101) in wild type. We obtained a similar result by immunostaining both wild type and *him-18* mutant gonads with a HIM-8 antibody, which specifically localizes to the pairing center end of the X chromosome [29] (Figure 2C and 2D and data not shown). Therefore, homologous pairing is normal for autosomes and the X chromosome in *him-18* mutants. We determined that axis morphogenesis was indistinguishable from wild type, as exemplified by the normal kinetics and pattern of localization of axis-associated proteins such as the meiosis specific cohesin REC-8 and the cohesin SMC-3 (Figure S4). We then examined the formation of the synaptonemal complex (SC), the proteinaceous scaffold that forms between fully paired and aligned homologous chromosomes during meiosis. Immunostaining for SYP-1, a SC central region protein, revealed a SYP-1 localization between paired homologous chromosomes that is indistinguishable from wild type both at the transition zone and pachytene (Figure 2 and data not shown). Taken together, these results indicate that events occurring upon entrance into meiosis, such as homologous pairing, axis morphogenesis and synapsis do not require HIM-18.

### SPO-11–Dependent and –Independent Recombination Intermediates Accumulate in *him-18* Mutants

**Figure 3:**
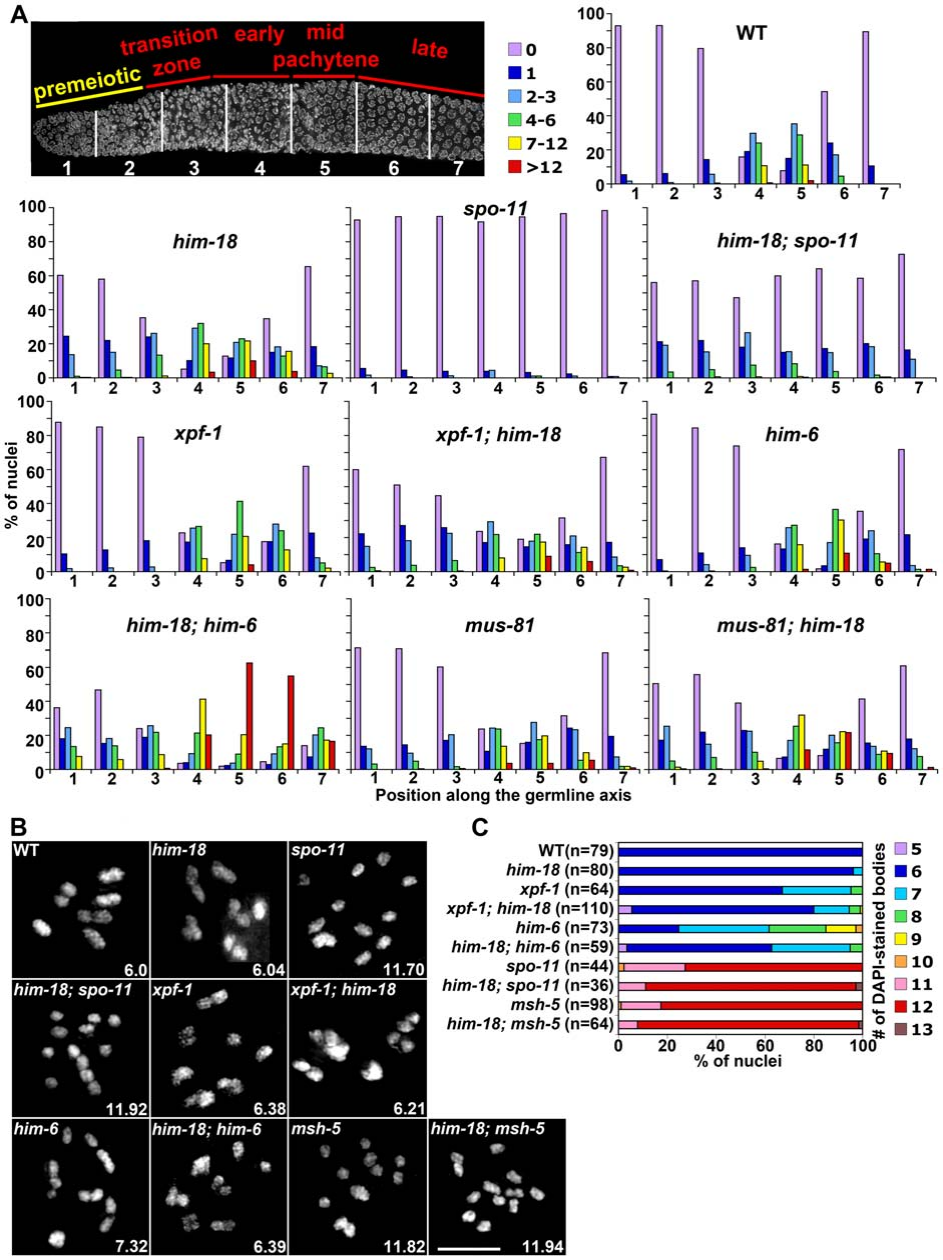
HIM-18 is required for DNA repair in both mitotic and meiotic germ cells. (A) Histograms depict the quantitation of RAD-51foci in germlines of the indicated genotypes. The number of RAD-51 foci per nucleus is categorized by the color code shown on the right. The percent of nuclei observed for each category (y-axis) are depicted for each zone along the germline axis (x-axis). 5–10 gonads were scored in each genotype. (B) High magnification images of DAPI-stained bodies in the diakinesis oocyte just before the spermatheca (-1 oocyte). The average number of DAPI-stained bodies is shown at the bottom right of each panel. Bar, 5 µm. (C) Quantitation of DAPI-stained bodies (including fragments). n = number of diakinesis nuclei scored for each genotype.

To investigate whether the defects in meiotic chromosome segregation observed in *him-18* mutants reflect defects in DSB repair, we performed a quantitative comparison of the levels of RAD-51 foci in the germlines of both wild type and *him-18* mutants (Figure 3A and Figure S5). Since nuclei are positioned in a temporal-spatial gradient throughout the germline in *C. elegans*, proceeding in a distal to proximal orientation from mitosis into the various stages of meiotic prophase I, levels of RAD-51 foci were assessed both in mitotic (zones 1 and 2) and meiotic (zones 3–7) nuclei (Figure 3A and 3B, Figure S5, S6, S7, S8 and [30]). In wild type, only a few mitotic RAD-51 foci were observed at zones 1 and 2 (5.8% and 1.2% of nuclei contained 1 and 2–3 RAD-51 foci, respectively; Figure 3A and Figure S5). In these mitotic nuclei, RAD-51 foci are thought to be mainly derived from single-stranded DNA gaps formed at stalled replication forks or resected DSBs resulting from collapsed replication forks [31]. During meiotic prophase, as a result of SPO-11-dependent programmed meiotic DSB formation, levels of RAD-51 foci start to rise at the transition zone (zone 3), then accumulate maximally at early to mid-pachytene (zones 4 and 5; nearly 90% of nuclei contain an average of 3.3 RAD-51 foci), and are reduced at late pachytene (zones 6 and 7) [30]. In *him-18* mutants, levels of RAD-51 foci were higher than those observed in wild type germlines, both in mitotic (23%, 14% and 3% of nuclei contained 1, 2–3 and 4–6 RAD-51 foci, respectively, in zones 1 and 2; *P*<0.0001 for both zones) and meiotic nuclei (an average of 5 RAD-51 foci/nucleus in zones 4 and 5; *P*<0.0001 and *P* = 0.0021, respectively) (Figure 3A and Figure S5). Moreover, higher levels of RAD-51 foci persisted through late pachytene in *him-18* mutants compared to wild type (3.2 RAD-51 foci/nucleus compared to 0.9, *P*<0.0001, and 0.9 foci/nucleus compared to 0.1, *P* = 0.0001, in zones 6 and 7, respectively) suggesting either a delay in meiotic DSB repair or an overall increase in the levels of DSBs formed during meiosis.

**Figure 4:**
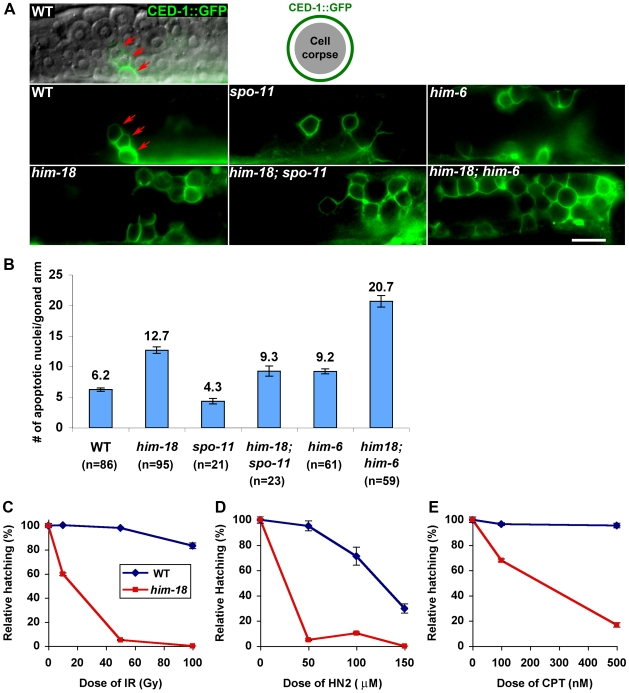
*him-18* mutants show increased germ cell apoptosis and sensitivity to multiple types of DNA damage. (A) High magnification differential interference contrast (DIC) and fluorescent images of CED-1::GFP encircling apoptotic nuclei in the late pachytene region of the germline. Arrows indicate CED-1::GFP signals. (B) Quantitation of germline apoptosis. Apoptotic corpses visualized in CED-1::GFP transgenic animals were scored. n = number of gonad arms scored for each genotype. (C–E) Relative hatching of wild type and *him-18* mutants after treatment with the indicated doses of (C) ionizing radiation (IR), (D) nitrogen mustard (HN2), and (E) camptothecin (CPT). Hatching is plotted as a fraction of the hatching observed in untreated animals. Error bars indicate standard error of the mean for 20 animals in each of two independent experiments.

Interestingly, larger RAD-51 foci were observed throughout both mitotic and meiotic nuclei in *him-18* mutants compared to wild type (Figure S8), some of which were still present in pachytene nuclei in *him-18; spo-11* double mutants. However, these SPO-11-independent RAD-51 foci failed to result in physical attachments between homologs (chiasmata). Specifically, instead of six bivalents, corresponding to the six pairs of homologous chromosomes held together by chiasmata, observed in DAPI-stained diakinesis oocytes in wild type, an average of 11.9 DAPI-stained bodies were observed in *him-18; spo-11* double mutants (n = 36), similar to *spo-11* single mutants (11.7 DAPI-stained bodies; n = 44) (Figure 3B and 3C). We also observed elevated levels of germ cell apoptosis in both *him-18* and *him-18; spo-11* double mutants, compared to wild type (*P*<0.0001 and *P* = 0.0009, respectively) and *spo-11* mutants (*P*<0.0001, respectively; Figure 4A and 4B). Furthermore, the elevated germ cell apoptosis in both *him-18* and *him-18;spo-11* mutants was suppressed following depletion of *cep-1/p53* by RNAi (Figure S2C and Figure S9), suggesting that the elevated apoptosis stems from the activation of the DNA damage checkpoint in late pachytene as a result of damage incurred both during mitosis and meiosis. Altogether, these observations suggest that SPO-11-independent mitotic RAD-51 foci persist into pachytene and that these unresolved recombination intermediates contribute in part to the increase in germ cell apoptosis observed in *him-18* mutants. However, the increased RAD-51 foci and germ cell apoptosis observed in mid to late pachytene, also support a role for HIM-18 specifically in HR during meiosis and are not simply a result of DNA damage being carried over into meiosis from defects in mitosis. This is further supported by the observation that events occurring upon entrance into meiosis were indistinguishable from wild type, and that despite homologous pairing and synapsis, repair of SPO-11-dependent DSBs was impaired.

### Loss of HIM-18 Confers DNA Damage Sensitivity

To gain further support for a role for HIM-18 in DNA damage repair, young adult hermaphrodites were exposed to different types of DNA damage and embryonic viability was monitored as an index of sensitivity (see Experimental Procedures). Embryonic viability after DNA damage treatment was plotted as a percentage of the hatching after DNA damage normalized by that in untreated *him-18* mutants. *him-18* mutants were hypersensitive to DSBs induced by γ-irradiation (IR). Only 60%, 5% and 0% hatching was observed in *him-18* mutants exposed to 10, 50 and 100 Gy, respectively, compared to wild type worms where a high level of hatching was observed even following the highest IR exposure level (83% at 100 Gy) (Figure 4C). Exposure to nitrogen mustard (HN2), which induces DNA interstrand crosslinks (ICLs) that obstruct essential cellular processes such as transcription and replication, resulted in significantly decreased hatching levels in *him-18* mutants (5% hatching at 100 µM HN2) compared to wild type (95% hatching at 100 µM HN2) (Figure 4D). To further examine the role of HIM-18 in responding to lesions that affect replication fork progression, worms were exposed to camptothecin (CPT), which inhibits the detachment of topoisomerase I from DNA, thus preventing DNA re-ligation at a single-strand nick, which in turn results in a single ended DNA double-strand break when collision of a replication fork occurs at the lesion [32]. Treatment with CPT resulted in a decrease in hatching in *him-18* mutants (17% hatching at 500 nM CPT) compared to wild type (96% at 500 nM CPT) (Figure 4E). In addition, both wild type and *him-18* mutants were examined following exposure to the ribonucleotide reductase inhibitor hydroxyurea (HU), which results in a checkpoint-dependent cell cycle arrest (Figure S10C). *him-18* mutants showed hypersensitivity to HU, further suggesting that *him-18/slx-4* is required to resolve stalled replication forks. Furthermore, the levels of RPA-1 foci were indistinguishable between both wild type and *him-18* germlines (Figure S10A), in contrast to the increase in RAD-51 foci observed at the premeiotic region in *him-18* mutants compared to wild type (Figure 3A and Figure S5). This observation suggests that the frequency of replication stalling is similar between wild type and *him-18* mutants, but that there is a defect in the recovery from stalled replication forks in *him-18* mutants. Mitotic germ cell nuclei with larger nuclear diameters are observed in *him-18* mutants compared to wild type even prior to HU treatment, further suggesting replication stress is occurring in this background (Figure S10B and Table S1). Following HU treatment, larger nuclear diameters were observed in mitotic germ cell nuclei in both wild type and *him-18* mutants, suggesting that the S phase checkpoint is intact in *him-18* mutants. Since the checkpoint is apparently intact, but reduced survival was still observed in *him-18* mutants, this implies a DNA repair rather than a checkpoint defect. Taken together, a drastic reduction in relative hatching frequencies was observed in *him-18* mutants compared to wild type following exposure to all four kinds of genotoxic agents. These results suggest that HIM-18 is required for DSB repair, ICL repair and recovery from replication fork collapse.

### *him-18* Is Synthetic Lethal with *him-6*, the *C. elegans BLM* Homolog

Budding yeast *SLX4* was first identified in a synthetic-lethal screen for genes that are essential in an *sgs1* mutant background [17]. Given that HIM-18 and Slx4 share sequence homology and the analysis of DSB repair progression and DNA damage sensitivity suggest a functional conservation, we investigated whether *him-18* mutants show synthetic lethality with loss of *him-6*,the *C. elegans* homolog of *BLM*, the Bloom syndrome helicase gene [33]. The total number of eggs laid either by *him-18* or *him-6* single mutants were only moderately reduced compared to wild type (66% and 79% of wild type levels were observed, respectively), and among those eggs laid, 20% and 40%, respectively, hatched (Table 1). In contrast, *him-18;him-6* double mutants showed a drastic reduction in brood size (only 3% of wild type) and all the eggs laid failed to hatch (Table 1). To further investigate whether the increased embryonic lethality observed in the *him-18;him-6* double mutants correlated with defects in DSB repair, we quantified the levels of RAD-51 foci in their germlines (Figure 3A and Figure S5). Indeed, both mitotic and meiotic RAD-51 foci levels were drastically increased in *him-18;him-6* double mutants compared to either single mutant. On average, a 2.7- and 19-fold increase in RAD-51 foci/nucleus was observed in mitotic nuclei at zone 1 in *him-18;him-6* double mutants compared with *him-18* and *him-6* single mutants, respectively (*P*<0.0001), and a 2.9- and 2.3-fold increase in meiotic mid-pachytene nuclei at zone 5 (*P* = 0.0001) (Figure S6). Furthermore, germ cell apoptosis was elevated nearly two-fold in *him-18;him-6* mutants compared to either single mutant (*P*<0.0001, respectively; Figure 4A and 4B). We also detected thin DAPI-stained threads (hereafter referred to as chromatin bridges), frequently stained with RAD-51, connecting nuclei at the premeiotic tip in *him-18;him-6* mutants. Specifically, 9 out of 11 gonads contained pairs of nuclei connected by chromatin bridges. Chromatin bridges were observed in 8.6% (n = 19/221) of nuclei in zone 1. Moreover, between 1 and 25 RAD-51 foci were observed on 95% of these chromatin bridges (Figure S11). These data suggest that accumulation of unresolved toxic recombination intermediates results in synthetic lethality in *him-18;him-6* double mutants.

### HIM-18 and MUS-81 Have Additive Roles in DNA Repair

Mus81 has been shown to be required for meiosis in fission yeast [8],[34],[35] and has been implicated in HJ processing in eukaryotic cells [8],[9]. To determine whether it plays a role with HIM-18 in DNA repair in the *C. elegans* germline, we examined phenotypes suggestive of errors in chromosome segregation and the levels of RAD-51 foci in *mus-81* and *mus-81;him-18* double mutants (Table 1, Figure 3A, and Figures S6, S7, S8). The total number of eggs laid by *mus-81* mutants was reduced compared to wild type (47% of wild type levels), and 20.7% of the eggs laid failed to hatch. This embryonic lethality was not accompanied by an increase in the frequency of males among the surviving progeny, suggesting that MUS-81 is not required for the proper disjunction of the X chromosome at meiosis I and in agreement with [36]. In *mus-81;him-18* mutants embryonic lethality, but not the incidence of male progeny, was elevated compared to *him-18* single mutants (87.8% of the eggs laid failed to hatch; 5.6% males; *P*<0.0001 and *P* = 0.2272, respectively, by the Fisher's Exact test). Levels of RAD-51 foci in *mus-81* mutants were only increased in nuclei in the premeiotic region compared to wild type (levels of RAD-51 foci were similar during pachytene). However, levels of RAD-51 foci were increased in both premeiotic and pachytene nuclei in *mus-81;him-18* double mutants compared to either single mutant (Figure 3A, Figure S5, S6, S7, S8). Therefore, while MUS-81 may not play a critical role during meiosis in *C. elegans*, most of the *him-18* phenotypes are aggravated by *mus-81*. These results suggest that MUS-81 may have additive roles with HIM-18 during repair both in mitosis and meiosis.

### HIM-18 Interacts with SLX-1 and XPF-1

**Figure 5:**
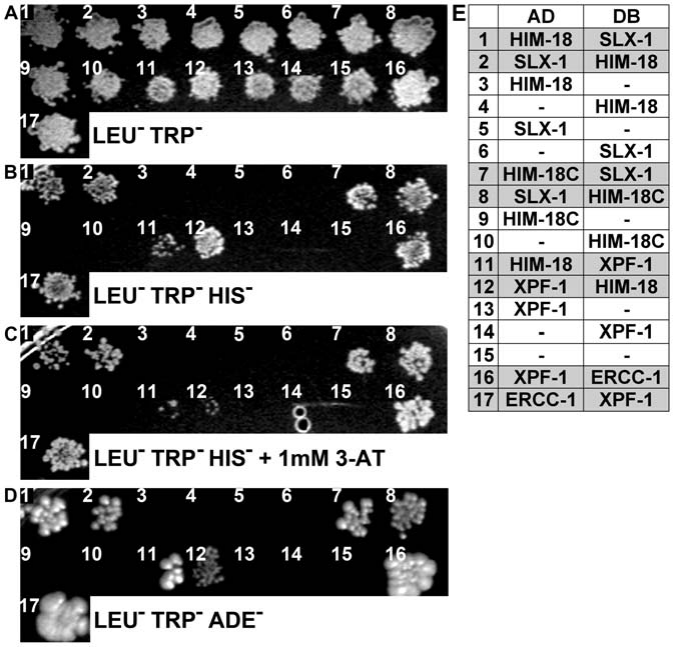
HIM-18 interacts with SLX-1 and XPF-1. The yeast two-hybrid system was used to examine the protein interactions between HIM-18, SLX-1, XPF-1 and ERCC-1. Both full length and truncations of HIM-18 were examined. Proteins were fused to either the DNA binding domain (DB) or the activation domain (AD) of GAL4. (A–D) Interactions were scored by growth on SC-Leu-Trp-His plates (B), growth on SC-Leu-Trp-His+1mM 3-AT plates (C), or growth on SC-Leu-Trp-Ade plates (D). (E) Matrix indicates the pair-wise combinations for AD-X and DB-Y interactions examined at each position on the plates. Positive interactions are shaded in gray.

Slx4 interacts with Slx1, Rad1/XPF, Rtt107/Esc4 and Cdc27 in *S. cerevisiae* [17], [37]-[39]. In *D. melanogaster*, MUS312 interacts with MEI-9/XPF [16]. To determine whether HIM-18 interacts with SLX-1, XPF-1 or ERCC-1, which forms a heterodimer with XPF-1 in *C. elegans* [40], we tested the full length and various regions of HIM-18 for interactions with these proteins using the yeast two-hybrid system (Figure 5). We divided HIM-18 into three parts, namely HIM-18N (amino acids 1 to 166), HIM-18M (amino acids 165 to 437), and HIM-18C (amino acids 420 to 718). HIM-18N contains the zinc finger domain, HIM-18M contains the coiled-coil and BTB domains, and HIM-18C contains the SAP and leucine zipper motifs. DB-HIM-18M showed self-activation precluding further analysis with this construct. Both HIM-18 full length and HIM-18C interact with SLX-1 in either orientation in the yeast two-hybrid system indicating that SLX-1 binds to the C- terminal region of HIM-18. HIM-18 full length also interacts with XPF-1 in either orientation although this interaction is weaker than those observed between HIM-18-SLX-1 or XPF-1-ERCC-1. Similar to *D. melanogaster*, where an interaction between MUS312 and ERCC1 was not detected [16], we also failed to observe an interaction between HIM-18 and ERCC-1 (data not shown). We obtained similar results using different combinations of yeast strains and plasmids (Figure S12). Thus, HIM-18 physically interacts with SLX-1 and XPF-1 in *C. elegans*, similar to the interactions observed involving Slx4 in *S. cerevisiae* [17], and its orthologs in *S. pombe* [20] and *D. melanogaster* [16].

**Figure 6:**
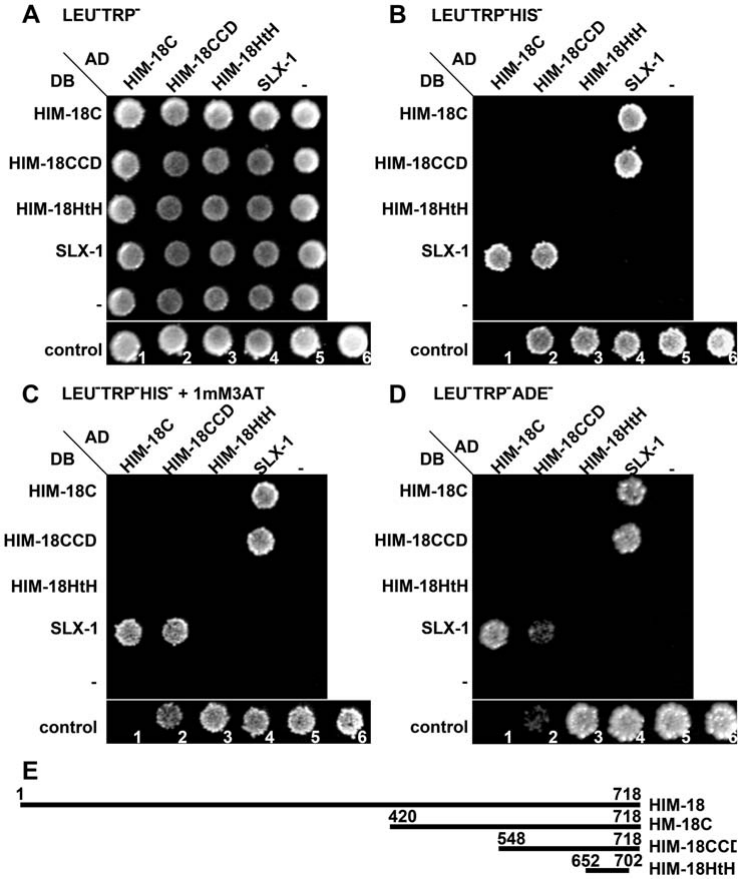
The conserved C-terminal domain (CCD) in HIM-18 is required for interaction with SLX-1. The yeast two-hybrid system was used to further refine the domain of HIM-18 required for the interaction with SLX-1. (A–D) Interactions were scored by growth on SC-Leu-Trp-His plates (B), growth on SC-Leu-Trp-His+1 mM 3AT plates (C), or growth on SC-Leu-Trp-Ade plates (D). Negative (No. 1) and positive (No. 2–6) controls are used as described in [79],[80]. (E) Schematic representation of the region of HIM-18 used in the yeast two-hybrid assay.

To further refine the region of HIM-18 required for the interaction with SLX-1, we specifically examined two domains contained within the C-terminus defined based on recent studies of the mammalian SLX4/BTBD12 protein [41]–[43]. Specifically, we examined the conserved C-terminal domain (CCD) [41],[42] and the helix-turn-helix (HtH) region contained within the CCD [43]. We observed that HIM-18CCD, but not HIM-18HtH, binds to SLX-1 (Figure 6). These results are in agreement with the analysis of the human SLX4/BTBD12 [41] [42].

### HIM-18 and XPF-1 Are Required for Normal Levels of Meiotic Crossovers

**Figure 7:**
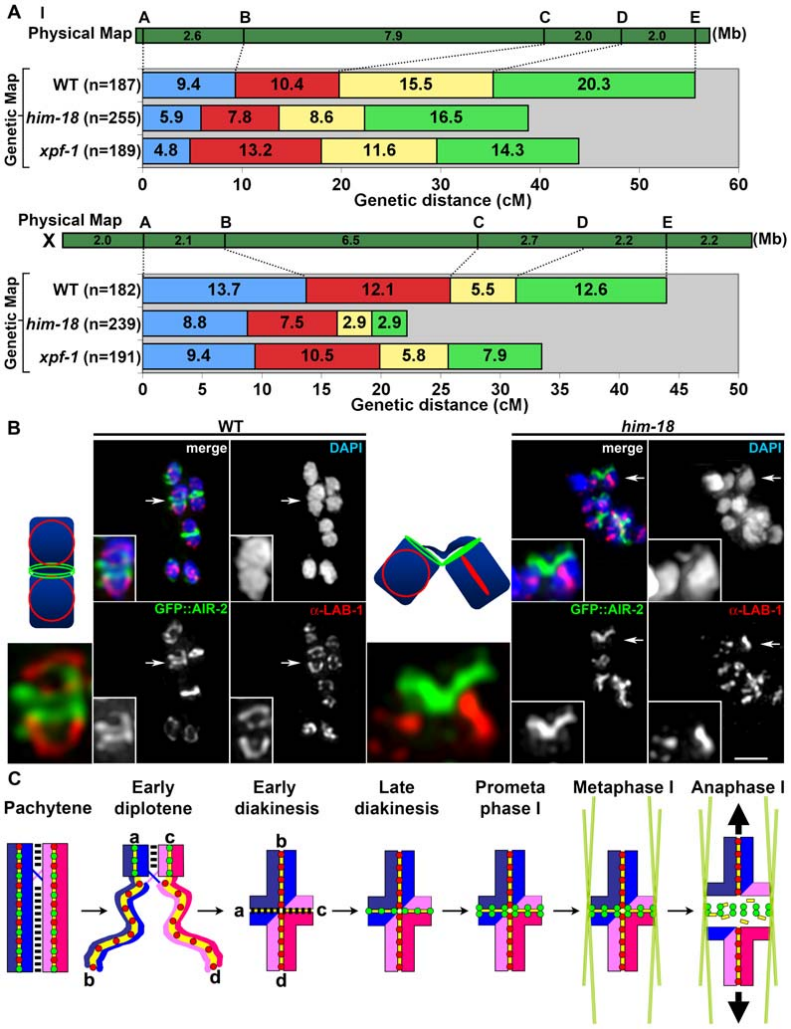
Crossover frequencies and bivalent stability at prometaphase I are reduced in *him-18* mutants. (A) Analysis of CO frequencies on chromosomes I (top) and X (bottom) in wild type, *him-18* and *xpf-1* mutants. Positions of SNP markers delimiting four intervals (A–B, B–C, C–D, and D–E) are shown along with the physical and genetic maps. n = number of cross-progeny scored. (B) Bivalents in prometaphase I oocytes in wild type and *him-18* mutants were examined for LAB-1 and GFP::AIR-2 localization to highlight both axes of this configuration. Arrows indicate the bivalents enlarged in the insets. The illustrations depict the chromosome axis configurations. Bar, 2 µm. (C) Diagram depicting chromosome behavior from pachytene to anaphase I. Paternal sister chromatids are in blue and maternal sister chromatids are in red. Crossover recombination (indicated by the X) is completed in the context of fully synapsed homologous chromosomes in pachytene. Asymmetric disassembly of the SC, exemplified by SYP-1 in this diagram, starts in late pachytene and progresses through early diplotene where SYP-1 is still observed in regions where homologs remain coaligned, but is no longer present in homologue segments that are disassociating. The region where SYP-1 is still present corresponds to the short arm of the bivalent at early diakinesis, which is later occupied by AIR-2, whereas LAB-1 localizes on the long arm. The short and the long arms are defined by the off-center position of the single crossover event that occurs between each pair of homologs. This late prophase chromosome remodeling process around the off-center crossover reveals the chiasma. To better understand the conformational change undergone by the bivalents from diplotene to diakinesis, the end of each chromosome is labeled a, b, c, and d, respectively. Bivalents align at the metaphase I plate (the long arms are positioned perpendicular to the metaphase plate) and this involves microtubules and the motor activity of chromokinesin at the short arm (not shown). At anaphase I, AIR-2 is proposed to phosphorylate the cohesin REC-8 localized at the short arm (REC-8 localized at the long arm is protected by LAB-1) resulting in loss of sister chromatid cohesion at the short arm. The recombined homologs then segregate away from each other towards opposite poles of the meiosis I spindle (bold arrows). REC-8 (yellow bars), SYP-1 (black bars), LAB-1 (red circles), AIR-2 (green circles), and microtubules (green lines).

The *S. cerevisiae* Slx4-Slx1 complex can cleave branched DNA substrates such as HJs *in vitro* and both MUS312 and MEI-9/XPF in *D. melanogaster* have been implicated in HJ resolution. Since both *xpf-1(e1487)* mutants and *xpf-1(RNAi)* worms showed embryonic lethality (20.2%, n = 2348, and 7.8%, n = 2890, respectively), a high incidence of males (4.5% and 3.5%, respectively) (Table 1 and Figure S2B) and elevated levels of RAD-51 foci at late pachytene (zone 6) (3.1 and 2.1±0.29 foci/nucleus, respectively; *P*<0.0001 and *P* = 0.0028 by the two-tailed Mann-Whitney test; 95% C.I.) compared to wild type (0.9 foci/nucleus) and control (RNAi) (0.9±0.15 foci/nucleus) (Figure 3A and Figure S5), it is possible that XPF-1 may also play a role in meiotic recombination in *C. elegans*. Given that meiotic COs require HJ resolution, we assessed the role of HIM-18 and XPF-1 in meiotic CO formation by comparing CO frequencies along both chromosomes I and X between wild type and mutants for *him-18* and its interaction partner *xpf-1* (Figure 7A and Table S2). We were precluded from performing this analysis for *slx-1* mutants due to the lack of an available strong loss-of-function mutant (T. Saito and M. Colaiácovo, unpublished results). On chromosome I, a 55.6cM interval corresponding to 96% of this chromosome's whole length (interval A to E) was assayed using 5 snip-SNPs. The CO frequency in this interval was reduced to 69.8% (*P* = 0.0005) and 79% (*P* = 0.0302) of wild type, respectively in *him-18* and *xpf-1* mutants. On chromosome X, a 44cM interval corresponding to 76% of this chromosome's whole length (interval A to E) was assayed using 5 snip-SNPs. The crossover frequency observed in this interval was reduced to 50.5% (*P*<0.0001) and 76.1% (*P* = 0.0434) of wild type, respectively in *him-18* and *xpf-1* mutants. As in wild type, double COs were not detected in either mutant for either chromosome, indicating that CO interference was not impaired. Finally, in wild type *C. elegans*, CO distribution is biased towards the terminal thirds of autosomes and is more evenly distributed along the X chromosome [44]. Our analysis suggests that these distribution patterns are not altered among the remaining COs observed in *him-18* and *xpf-1* mutants (the reduction in COs observed for interval D-E on the right end of the X chromosome in *him-18* mutants was not significant compared to wild type; *P* = 0.0553). Taken together, these data suggest that HIM-18 and XPF-1 do not play a role in CO positioning, but are required for normal levels of CO formation in the autosomes and the X chromosome.

### HIM-18 Acts Following Holliday Junction Formation during Meiosis

The fact that the frequency but not the position of meiotic crossover events is affected in *him-18* and *xpf-1* mutants suggests that HIM-18 and XPF-1 may be required very late in the process of crossover formation. To further assess this, we examined the genetic interaction between *him-18* and *msh-5* (Figure 3B and 3C and Table 1). MSH-5 and HIM-14/MSH-4 function downstream of the chromosomal association of the RAD-51 strand-exchange protein, but upstream of dHJ resolution during meiosis [30]. We observed approximately 12 DAPI-stained bodies in diakinesis oocytes in both *msh-5* and *him-18;msh-5* mutants, compared to the nearly 6 DAPI-stained bodies observed in both wild type and *him-18* mutants. These results suggest that HIM-18 may act downstream of MSH-5, after dHJ formation. However, until further studies address the nature of the bivalent connections observed in *him-18* mutants, we cannot exclude the possibility that HIM-18 may also act on a completely different pathway from MSH-5.

Given that CO recombination results in chiasmata that persist until the metaphase I to anaphase I transition during meiosis, it was intriguing that despite the reduction in meiotic CO recombination frequencies observed in *him-18* mutants, we mostly observed 6 pairs of attached bivalents in *him-18* diakinesis oocytes (Figure 3B and 3C). However, careful examination revealed that the morphology of the DAPI-stained bivalents observed in *him-18* mutants was distinct from wild type in diakinesis and prometaphase I, although this was more clearly apparent in the latter (Figure 7B and 7C). Specifically, homologs were loosely attached and 84% (n = 16/19) of the oocytes examined had at least one bivalent held together by a thin DAPI-stained thread at prometaphase I. The “fragility” of the connections between homologs is further highlighted by the localization of the LAB-1 and AIR-2 proteins on bivalents at prometaphase I. LAB-1 is a protein recently implicated in the protection of sister chromatid cohesion and is restricted to the longer axes of the bivalents from late prophase I through the metaphase I to anaphase I transition [45]. AIR-2 is the *C. elegans* Aurora B kinase and is restricted to the mid-bivalent from late diakinesis through metaphase I. However, in *him-18* mutants the mid-bivalent is observed separating prematurely at prometaphase I as indicated by the partially separated (V-shaped) AIR-2 ring-like signals in 68% of the oocytes examined (n = 13/19; *P*<0.0001 by the two-sided Fisher's Exact test, 95% C.I.) compared to 8.3% (n = 2/24) in wild type (Figure 7B). Taken together, these fragile attachments between homologs suggest that HIM-18 is required for chiasma formation and support the increased chromosome nondisjunction observed in *him-18* mutants.

### The Formation of Mature Bivalents Is Delayed in *him-18* Mutants

**Figure 8:**
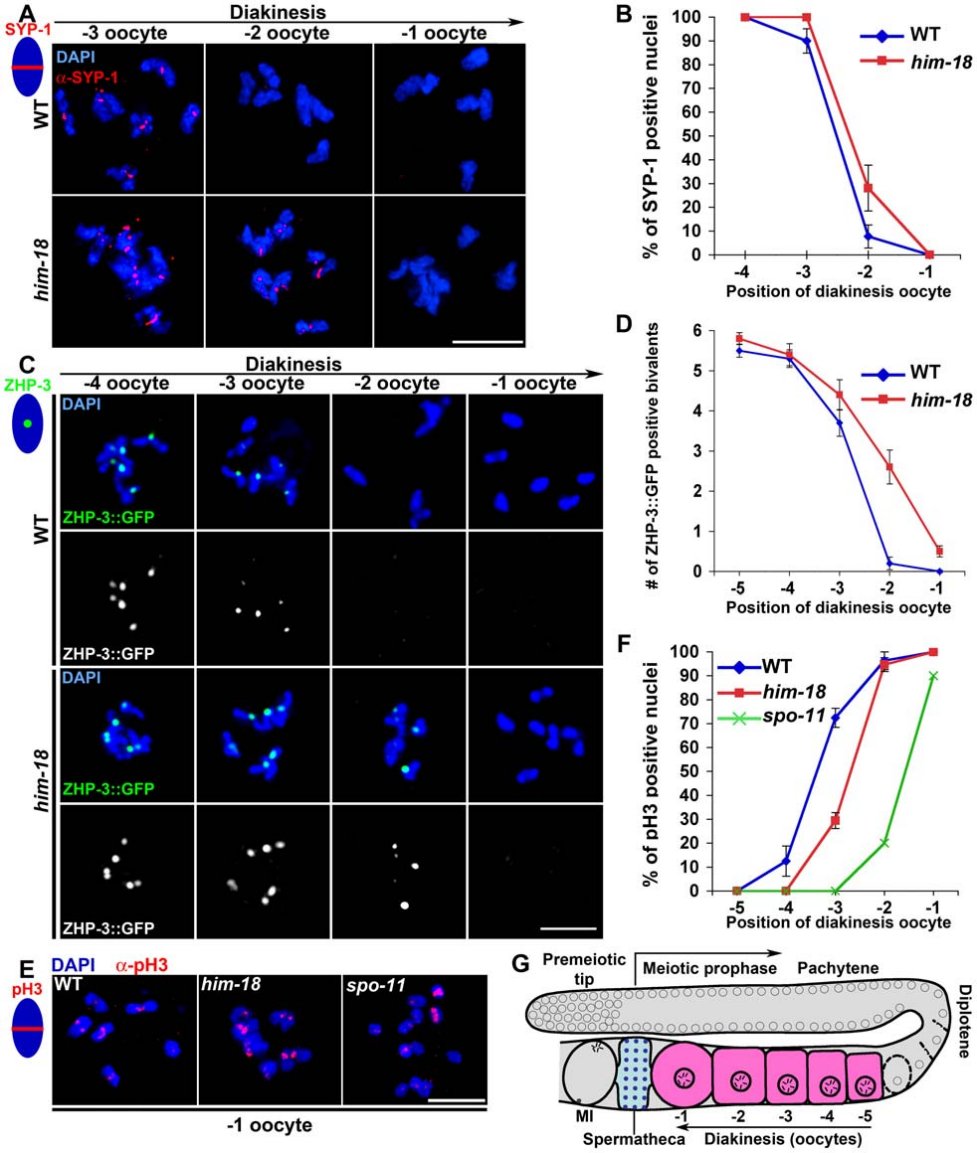
Delayed bivalent differentiation during diakinesis in *him-18* mutants. (A) SC disassembly, determined by SYP-1 immunolocalization, is delayed in *him-18* mutants compared to wild type. The three last oocytes in diakinesis are presented in a left to right orientation. Bar, 5 µm. (B) Quantitation of SYP-1 localization on DAPI-stained chromosomes in late diakinesis oocytes in wild type and *him-18* mutants (55 and 52 gonads scored for each genotype, respectively). (C) The ZHP-3::GFP foci marking nascent CO recombination sites persist longer on DAPI-stained chromosomes in *him-18* mutants compared to wild type. Bar, 5 µm. (D) Quantitation of ZHP-3::GFP foci in diakinesis oocytes of wild type and *him-18* mutants (32 and 26 gonads scored for each genotype, respectively). (E) Histone H3 phosphorylation (pH 3) on DAPI-stained chromosomes in –1 oocytes at diakinesis in the various indicated genotypes. Bar, 5 µm. (F) Quantitation of Histone H3 phosphorylation in late diakinesis oocytes in wild type (n = 36), *him-18* (n = 47) and *spo-11* (n = 10). (G) Schematic representation of one arm of the *C. elegans* gonad. Chromosome remodeling initiates in late pachytene and results in the formation of six bivalents at diakinesis, where they undergo cellularization and individualized single oocytes are observed. The oocyte closest to the spermatheca is referred to as the –1 oocyte. Diakinesis oocytes upstream from the –1 oocyte are labeled accordingly.

Crossovers or crossover precursors trigger chromosome remodeling in late meiotic prophase resulting in mature bivalent formation [46]. Therefore, to investigate whether HIM-18 affects the timing of mature bivalent formation, we assessed the kinetics of events correlated with chromosome remodeling in late prophase such as SC disassembly, the chromosomal dissociation of a CO recombination site marker and Histone H3 serine 10 phosphorylation (Figure 8). During SC disassembly, which initiates in late pachytene in wild type germlines, SC central region components that were previously localized throughout the full length of chromosomes become progressively restricted to the mid-bivalent by early diakinesis and are mostly gone from chromosomes by late diakinesis (only 10.9% of –2 oocytes carried 3 or more bivalents with residual SYP-1 signal, n = 55) (Figure 8A and 8G and [46]). In contrast, although early meiotic events proceed with normal kinetics in *him-18* mutants, SC disassembly is delayed in this background, as evidenced by the higher levels of –2 oocytes with chromosome-associated SYP-1 (28.8%, n = 52; *P*<0.0345) (Figure 8A and 8B). Recently, ZHP-3, a homolog of the budding yeast Zip3 protein, was suggested to mark CO recombination sites starting in late pachytene in *C. elegans* [47]. Interestingly, despite the reduction in CO frequencies observed in *him-18* mutants, we observed approximately six ZHP-3::GFP foci/nucleus in oocytes at early diakinesis (-5 oocytes) in both wild type (n = 25) and *him-18* mutants (n = 18) (Figure 8C and 8D). This suggests that ZHP-3 may mark a CO precursor instead of the mature CO during late pachytene through diakinesis. Moreover, the timing of dissociation of ZHP-3::GFP from chromosomes was delayed in a similar fashion to that of SC disassembly in *him-18* mutants (Figure 8C and 8D). While in wild type, between 5.3 to 3.7 ZHP-3::GFP foci/nucleus were observed until mid-diakinesis (–4 and –3 oocytes, n = 29 and 32, respectively) and were mostly no longer detected by late diakinesis (0.2 foci/-2 oocyte, n = 32), in *him-18* mutants, in average 2.6 ZHP-3::GFP foci (n = 26) were still present in the –2 oocytes (Figure 8C and 8D). Finally, quantitative analysis of Histone H3 phosphorylation (pH 3), a chromosomal substrate of AIR-2 kinase [45],[48],[49], indicated that the appearance of nuclei with pH 3 positive chromosomes is delayed in *him-18* mutants compared to wild type in late diakinesis (30% of –3 oocytes in *him-18* mutants carried pH 3 positive chromosomes compared to 72% in wild type) (Figure 8E and 8F). Interestingly, CO-defective *spo-11* mutants in which, similar to *him-18* mutants, events occurring upon entrance into meiosis such as chromosome pairing and synapsis are normal [50], showed the most delay in Histone H3 phosphorylation, further implicating mature CO formation as a requirement for proper timing of Histone H3 phosphorylation. Taken together, these data suggest that chromosome remodeling during late prophase is delayed in *him-18* mutants due to impaired CO formation.

## Discussion

### HIM-18 Is a New Late HR Intermediate Processing Factor Conserved Among Eukaryotes

**Figure 9:**
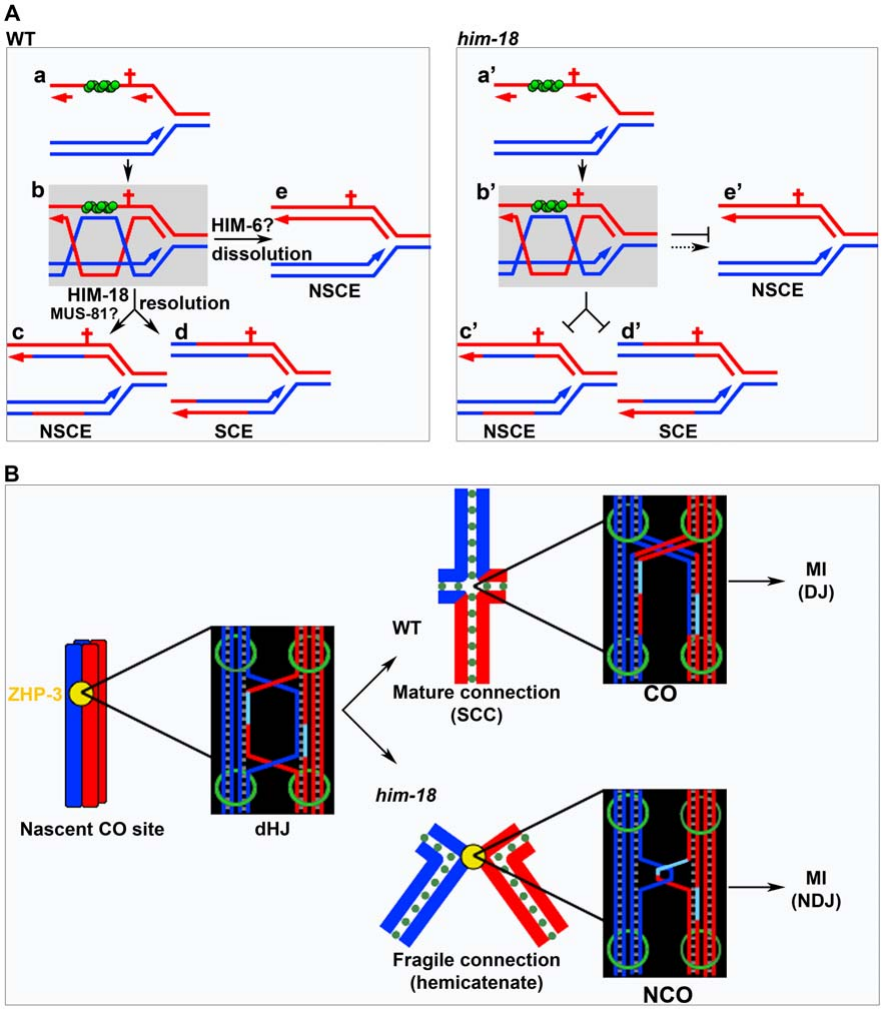
HIM-18 facilitates the processing of dHJs promoting restart of stalled replication forks and formation of meiotic COs. (A) Replication restart. In wild type, both endogenous and exogenous replication fork barriers (red cruciform) can stall replication fork progression. For simplicity, only a lesion at the lagging strand is shown. (A-a) RAD-51 is loaded on a single-strand gap. (A-b) RAD-51-mediated dHJ formation. dHJ is processed by HIM-18 and possibly MUS-81, resulting in non-sister chromatid exchange (NSCE) (A-c) and SCE (A-d). (A-e) Dissolution pathway mediated by HIM-6 (and TOP-3) results in NSCE. In *him-18* mutants, dHJ processing is impaired (A-c' and A-d') and dissolution is impaired and/or delayed (A-e'). (B) Meiotic CO formation. Paternal sister chromatids are blue and maternal sister chromatids are red. ZHP-3 marks the nascent CO site during late pachytene. In wild type, HIM-18-dependent dHJ processing results in the formation of a chiasma. Sister chromatid cohesion (SCC) contributes to the tension promoting the proper alignment of the bivalent at the metaphase I plate. Disjunction (DJ) of the homologous chromosomes following degradation of SCC along the mid-bivalent region occurs at anaphase I (MI). In *him-18* mutants, dHJ processing is impaired. Dissolution intermediates such as hemicatenane structures or the complete dissolution of the dHJ may manifest in chromosome bridges and lagging chromosomes observed at anaphase I resulting in chromosome non-disjunction (NDJ). Green circles in (A) represent RAD-51; green circles and rings in (B) represent cohesin.

Several groups reported that the Slx1-Slx4 complex cleaves HJs *in vitro*, although this cleaving activity is weak and inconsistent with an authentic HJ resolvase activity [7],[19],[20]. Although we do not know yet whether *C. elegans* SLX-1-HIM-18 has authentic HJ cleaving activity *in vitro*, our genetic and cytological data suggest that some HR intermediates are processed in a HIM-18-dependent manner. We did not identify any known nuclease motifs in HIM-18. However, we showed that two nucleases, SLX-1 and XPF-1, interact with HIM-18. SLX-1 is conserved from yeast to humans and contains an URI nuclease domain and a PHD finger domain. Although it has been reported that XPF-ERCC1 mainly cuts simple-Y, bubble, stem loop and 3′-flap structures in *S. cerevisiae* and *H. sapiens* [51], whether the substrate specificity of XPF-1 is altered from those reported DNA structures to HJs due to the interaction with HIM-18 during HR is unknown. Notably, the SAP motif of *S. pombe* Cce1, a mitochondrial HJ resolvase, is required for stable binding to HJs. An attractive hypothesis that builds on this observation is that HIM-18 may bind to the HJs via its SAP motif and promote the nuclease activity of SLX-1 and XPF-1. In this vein, HIM-18 could serve as a scaffold accommodating different interaction partners (nucleases), thereby facilitating the resolution of HJ intermediates arising in different biological contexts as depicted in our model (Figure 9). Specifically, we propose that HIM-18 function is required for replication restart after DNA damage and correct CO formation during meiosis to maintain genomic integrity in the germline.

### HIM-18 Function in Replication Restart

We showed that HIM-18 is required for the repair of DNA damage arising during DNA replication in the germline. When a replication fork collides either with a spontaneous or an artificial barrier, single-strand gaps (SSG) can be generated at either the lagging or leading strands (Figure 9A). To complete an error-free DNA replication, SSGs must be repaired by HR, and during mitosis, this involves the use of a sister chromatid, instead of a homologous chromosome, as a template for repair. We propose that HIM-18 is required for processing late HR intermediates after the RAD-51-mediated strand exchange and pairing (Figure 9A-c and 9A-d). It is conceivable that MUS-81 also functions in this step as it can cleave substrates mimicking dHJs in biochemical assays [52] and has been implicated in the repair of spontaneous DNA damage [36] and DNA ICLs [53]. Therefore, it will be interesting to test if HIM-18 and MUS-81 have overlapping functions in HJ processing during replication collapse. In *BLM*-deficient cells, sister chromatid exchange (SCE) is increased [54]. As BLM has been implicated in the dissolution of HJs to yield NCOs in multiple organisms [55],[56], the increased levels of chromatin bridges with numerous RAD-51 foci observed in *him-18; him-6* double mutants may represent an accumulation of repair intermediates during DNA replication stress, which are not processed either via HIM-18 or dissolution by HIM-6. While dHJ unwinding activity has not been reported for HIM-6 in *C. elegan*s, our data is consistent with distinct roles for HIM-6 and HIM-18 in processing dHJ intermediates (Figure 9A-e').

Apparently, *him-18* and *xpf-1* mutants are distinct from each other regarding repair of spontaneous DNA damage during mitosis. In contrast to *him-18* mutants, *xpf-1* mutants do not show an increase in the levels of RAD-51 foci in the premeiotic region of the germline and the RAD-51 staining pattern observed in *xpf-1;him-18* double mutants is very similar to that in *him-18* single mutants. XPF-1 is required for unwinding or repairing G4 DNA (G-quadruplex) structures during DNA replication in mutants of the *C. elegans* homolog of the FANCJ DNA helicase, *dog-1* [57],[58]. In contrast, we did not detect elevated levels of deletions on polyG/C-tracts in *dog-1;him-18* double mutants (Figure S13). Therefore, HIM-18 does not play a role in the unwinding of G4 DNA during replication. Likewise, whereas XPF-1 is required for NER [59], fly MUS312 and yeast Slx4, the HIM-18 orthologs, do not function in NER [16],[20]. Taken together, these observations suggest that HIM-18 has a distinct function from that of XPF-1 during mitotic proliferation.

### HIM-18 Function in Meiotic CO Formation

COs play a critical role in ensuring accurate meiotic chromosome segregation, as exemplified by the alterations in CO number and/or distribution frequently associated with human aneuploidies [60]. Ensuring the formation of at least one CO per homolog pair (obligate CO) is vital to the transmission of an intact genome during gametogenesis. In wild type *C. elegans*, one DSB per pair of homologous chromosomes engages in a CO pathway and is marked by ZHP-3. In *him-18* mutants, CO frequencies are decreased and offloading of ZHP-3 from nascent CO sites is delayed. This delay could be simply explained by dHJs being dissolved instead of resolved in *him-18* mutants and the dissolution process perhaps being time consuming (Figure 9B). Occasionally, however, dissolution is not completed in *him-18* mutants, because fragile connections (possibly hemicatenanes) are still detectable between homologs. This fragile connection persists through diakinesis into prometaphase I possibly leading to nondisjunction (NDJ) at anaphase I. This is further supported by our observation of chromosome bridges and lagging chromosomes at the metaphase I to anaphase I transition in *him-18* mutants (n = 9/15oocytes), in contrast to wild type where these were never detected (n = 0/17) (Figure S14). However, the presence of oocytes lacking either chromosome bridges or lagging chromosomes in *him-18* mutants suggests that at least a portion of the hemicatenanes may be finally dissolved by TOP-3 just prior to anaphase I. Taken together, our data are consistent with a role for HIM-18 in processing dHJs leading to CO formation during meiosis (Figure 9B).

*him-18;him-6* double mutants are synthetic lethal. Interestingly, with regard to the numbers of DAPI-stained bodies in oocytes at diakinesis, *him-18* suppresses the *him-6* mutation (*P*<0.0001, by the two tailed Mann-Whitney test; 95% C.I.). To explain this meiotic phenotype we propose that HIM-6 may play a role in stabilizing the meiotic D-loop and dHJ intermediates, perhaps via its helicase activity, and specifically promote a CO outcome. In *him-18;him-6* mutants, there is no HIM-6-dependent stabilization of D-loops. Some unstable D-loops may be processed via the SDSA pathway. The remaining D-loops may become unstable dHJs, which are not cleaved by HIM-18 and persist during diakinesis. Therefore, the number of DAPI-stained bodies in *him-18;him-6* mutants (6.39) is lower than that observed in *him-6* mutants (7.32) and higher than in *him-18* mutants (6.04).

A requirement for XPF in meiotic CO formation had only been observed, thus far, in *D. melanogaster*, where MEI-9 is essential for meiotic COs. Our genetic and cytological analyses suggest that XPF-1 seems to function in CO formation during *C. elegans* meiosis. Interaction of HIM-18 with XPF-1 may be important for altering the substrate-specificity of XPF-1 towards HJs during meiosis. We observed that the decrease in CO frequency on chromosome X in *xpf-1* mutants is milder than that in *him-18* mutants (*P* = 0.0120). These data suggest that HIM-18 may define yet another HJ processing activity distinct from XPF-1. Mus81, which is also known as a meiotic HJ resolvase, is specialized in interference-independent COs in yeast, plants and mammals [61]. However, in *C. elegans*, virtually all COs are HIM-14/MSH-4 and MSH-5-dependent, and therefore interference-dependent [62],[63]. Additionally, *mus-81* mutants are largely viable [36] suggesting that MUS-81 may not be required for meiotic COs in *C. elegans.* Further experiments will address whether SLX-1, which we demonstrated is a HIM-18 interaction partner, functions during meiotic CO formation.

### HIM-18 Expression May Undergo Post-Transcriptional Regulation

HIM-18 is expressed in the *C. elegans* germline, being enriched at the premeiotic tip and in late meiotic prophase (from late pachytene to diakinesis). HIM-18 protein levels, as detected by immunostaining, are low between the transition zone and the mid-pachytene stage. In contrast, analysis of mRNA expression by in situ hybridization (Y. Kohara, personal communication) suggests a more uniform pattern of expression from transition zone until diakinesis. These data suggest tight regulation of HIM-18 at the protein level, either by translational repression and/or protein degradation between transition zone through the mid-pachytene stage. The translation of a number of mRNAs in the *C. elegans* germline is repressed by GLD-1, a member of the STAR KH-domain family of RNA binding proteins [64]. However, sequence analysis revealed that HIM-18 harbors a destruction box (D-box; RXXL), an APC/C recognition motif and an Ubc9 recognition motif (ψKXE). These motifs are usually required for the ubiquitin- and SUMO-dependent proteolytic pathways. In *S. cerevisiae* Slx4 interacts with the APC/C component Cdc27, although it remains to be determined whether Slx4 is degraded by APC/C. Further analysis will reveal whether HIM-18 may be a target for a proteolytic pathway during transition zone to mid-pachytene.

In summary, we identified HIM-18 as an ortholog of yeast Slx4, fly MUS312 and mammalian BTBD12 proteins, which plays a critical role in the germline during DSB repair upon replication fork collapse in mitosis and SPO-11-dependent programmed meiotic DSB formation. Our results therefore identified HIM-18 as a new HJ processing factor in *C. elegans,* which is distinct from the Mus81-Eme1 complex.

## Materials and Methods

### C. elegans Genetics

*C. elegans* strains were cultured at 20°C under standard conditions [65]. The N2 Bristol strain was used as the wild-type background. The following mutations and chromosome rearrangements were used in this study: LGI: *mus-81(tm1937), dog-1(gk10), hT2[bli-4(e937) let-?(q782) qIs48](I; III)*; LGII: *xpf-1(e1487), mIn1[dpy-10(e128) mIs14](II);* LGIII: *him-18(tm2181)*, *unc-32(e189)*, *sDf121, qC1[dpy-19(e1259) glp-1(q339) qIs26] (III)*; LGIV: *spo-11(ok79)*, *him-6(ok412), msh-5(me23), nT1[ unc-?(n754) let-? qIs50] (IV; V)*, *nT1[qIs51] (IV; V)* [50], [62], [65]–[68].

### Analysis of HIM-18 Protein Conservation and Motifs

HIM-18 homology searches were performed using the Ensembl genome browser (http://www.ensembl.org/index.html) and Pfam (http://pfam.sanger.ac.uk/). Although the HIM-18 ortholog in *S. cerevisiae* was not identified by the Ensembl program, Pfam predicted that the SAP motif of HIM-18 is similar to that in yeast Slx4. The following motif prediction programs were applied to HIM-18 and its orthologs: COIL and P-SORT II for coiled-coil and leucine zipper predictions, Pfam and HHpred for zinc finger and BTB domain predictions [69]-[72].

### Antibody Preparation, DAPI Analysis, Immunostaining, and FISH

Rabbit anti-HIM-18 antibody was produced using a HIS-tagged fusion protein expressed from plasmid pDEST17 (Invitrogen) containing coding sequence corresponding to the first 166 amino acids of HIM-18. 6xHis-HIM-18N was expressed in BL21 *E. coli* cells and purified with the Ni-NTA Purification System (Invitrogen). Animals were immunized and bled by Sigma-Genosys, The Woodlands, TX. The antisera were affinity-purified against the 6xHis-HIM-18N peptide as described in [73].

Whole mount preparation of dissected gonads, DAPI-staining, immunostaining and analysis of meiotic nuclei were carried out as in [30] and [45], with the exception of the rabbit anti-HIM-18 antibody, where gonads were fixed with 1% formaldehyde for 5 minutes, then incubated in ice-cold methanol for 1 minute, followed by blocking with 1% BSA for 1 hour. Primary antibodies were used at the following dilutions: mouse α-RAD-51 (1∶100), rabbit α-SYP-1 (1∶100), guinea pig α-SYP-1 (1∶100), rabbit α-HIM-8 (1∶100), rabbit α-HIM-18 (1∶5000), rabbit α-LAB-1-Cy3 (1∶50), rabbit α-pH 3 (1∶100), mouse α-REC-8 (1∶100), rat α-SMC-3 (1∶100) and rabbit α-RPA-1 (1∶500).

FISH was performed as in [74] utilizing a probe to the 5S rDNA locus on chromosome V prepared as in [50].

### Time Course Analysis for RAD-51 Foci

Quantitative analysis of RAD-51 foci was performed as in [30] except that all seven zones composing the germline were scored. 5–10 germlines were scored for each genotype. The average number of nuclei scored per zone for a given genotype was as follows, ±standard deviation: zone 1, n = 215±48; zone 2, n = 259±57; zone 3, n = 246±61; zone 4, n = 217±41; zone 5, n = 190±34; zone 6, n = 154±24; zone 7, n = 137±31. Statistical comparisons between genotypes were performed using the two-tailed Mann-Whitney test, 95% confidence interval (C.I.).

### DNA Damage Sensitivity Experiments

Young adult *him-18/him-18* animals were picked from the progeny of *him-18/qC1* parent animals. To assess ionizing radiation (IR) sensitivity, animals were treated with 0, 10, 50 or 100 Gy of IR from a Cs^137^ source at a dose rate of 2.16 Gy/min. For nitrogen mustard (HN2) sensitivity, animals were treated with 0, 50, 100 or 150 µM of HN2 (mechlorethamine hydrochloride; Sigma) in M9 buffer containing *E. coli* OP50 with slow shaking in the dark for 19 hours. Treatment with camptothecin (CPT; Sigma) was similar, but with doses of 0, 100 or 500 nM. Following treatment with HN2 or CPT, animals were washed twice with M9 containing TritonX100 (100 µl/L) and plated to allow recovery for 3 hours. For hydroxyurea (HU) sensitivity, animals were placed on seeded MYOB plates containing 0 or 40 mM HU for 24 hours. HU sensitivity was assessed in 20 animals from 4–22 hours after HU treatment. For all other damage sensitivity experiments, 20 animals were plated 5 per plate and hatching was assessed for the time period 22–26 hours following treatment. Each damage condition was replicated at least twice in independent experiments.

### Quantitative Analysis of Germ Cell Apoptosis

22–24 hour post-L4 hermaphrodites expressing CED-1::GFP (mammalian MEGF10; [75]) were mounted under coverslips in 5 µl of a 15 mM sodium azide solution on 1.5% agarose pads. Apoptotic cells surrounded by CED-1::GFP signal were observed in the late pachytene region of the germline with a Leica DM5000 B fluorescence microscope. Between 21 and 95 gonads were scored for each genotype. Statistical comparisons between genotypes were performed using the two-tailed Mann-Whitney test, 95% C.I.

### Determining Crossover Frequencies

Meiotic CO frequencies were assayed utilizing single-nucleotide polymorphisms (SNP) markers as in [76], except that +/+ worms were used as a control. PCR and *Dra*I restriction digests of single worm lysates were performed as described in [77]. The following *Dra*I SNP primers were utilized: A (snp_F56C11), B (pkP1052), C (CE1-247), D (uCE1-1361), E (snp_Y105E8B) for chromosome I, and A (pkP6143), B (pkP6105), C (snp_F11A1), D (pkP6132), E (uCE6-1554) for the X chromosome. Statistical analysis was performed using the two-tailed Fisher's Exact test, 95% C.I.

### Yeast Two-Hybrid Analysis

Yeast two-hybrid was performed according to [78]. cDNA of HIM-18 full length, HIM-18N^1–166^, HIM-18M^165–437^, HIM-18C^420–718^, SLX-1 (open reading frame F56A3.2) full length, XPF-1 full length, and ERCC-1 (open reading frame F10G8.7) full length were cloned into the Gateway donor vector pDONR223. Each construct was then subcloned into 2 µ Gateway destination vectors pVV213 (activation domain (AD), *LEU2*+) and pVV212 (Gal4 DNA binding domain (DB), *TRP1*+). AD-Y and DB-X fusions were transformed into *MAT*a Y8800 and *MAT*α Y8930 yeast strains, respectively. These yeast strains have three reporter genes: *GAL2-ADE2*, *met2::GAL7-lacZ* and *LYS2::GAL1-HIS3*. *MAT*a Y8800 and *MAT*α Y8930 were mated on YPD plates and diploids carrying both plasmids were selected on SC-Leu-Trp plates. Similarly, we made pVV213/pVV212-containing AH109/Y187 and Mav203/Mav103 diploids and pDEST22/pDEST32-containing Y8800/Y8930 diploids (Figure S12). Pair-wise interactions were tested by scoring for yeast two-hybrid phenotypes (LacZ, -Ade, -His, -His+either 1 mM or 20 mM 3AT) at 30°C.

## Supporting Information

Figure S1 Immunostaining indicates lack of HIM-18 signal on the germline of *him-18* mutants. Low magnification image of a whole mounted *him-18* gonad where DAPI-stained chromosomes (blue) were immunostained with α-HIM-18 (red). Bar, 20 µm.
(7.73 MB TIF)

Figure S2 RT-PCR analysis of RNAi experiments. (A) RT-PCR in *him-18(RNAi)* worms compared to control (RNAi) worms. Feeding vector (pL4440) alone is the control (RNAi) indicated by ‘cont.’. *myo-3* expression was used as a loading control. (B) RT-PCR in *xpf-1(RNAi)* worms. *sgo-1* expression was used as a loading control. (C) RT-PCR in *cep-1(RNAi)* and *cep-1(RNAi);him-18* worms. *sgo-1* expression was used as a loading control. Each lane contains the RT-PCR products from single adult worms 22–24 hours post-L4.
(1.14 MB TIF)

Figure S3 Quantitative analysis of homologous pairing in *him-18* mutants. Quantitation of homologous chromosome pairing at the 5S rDNA locus on chromosome V. Graph depicts the percentage of nuclei carrying paired homologous chromosomes (y-axis) within each zone along the germline (x-axis). Homolog pairing levels are indistinguishable between wild type and *him-18* mutants throughout meiotic prophase. Data was collected from wild type (n = 3) and *him-18* (n = 4) whole mounted germlines examined by FISH. The average numbers of nuclei scored per zone for each genotype are as follows: zone 1, n = 101; zone 2, n = 135; zone 3, n = 125; zone 4, n = 113; zone 5, n = 104; zone 6, n = 81; zone 7, n = 65.
(0.34 MB TIF)

Figure S4 Axis morphogenesis is normal in *him-18* mutants. Low magnification image of whole mounted wild type and *him-18* gonads. DAPI-stained chromosomes (blue) were immunostained with α-REC-8 (green) and α-SMC-3 (red). The yellow dotted lines indicate the borders between the premeiotic tip (pmt) and the transition zone (tz). The direction of meiotic progression is indicated by the yellow arrow. Bar, 20 µm.
(9.99 MB TIF)

Figure S5 HIM-18 is required for DNA repair in both mitotic and meiotic germ cells. Low magnification images of germlines of the indicated genotypes immunostained with RAD-51 and DNA counterstained with DAPI. The seven zones in which RAD-51 foci are quantitated are depicted on the wild type germline. Bar, 20 µm.
(9.14 MB TIF)

Figure S6 Mean number of RAD-51 foci per nucleus. Quantitative analysis of RAD-51 foci depicted in Figure 3, is represented here as the mean number of RAD-51 foci observed per nucleus (y-axis) on each zone along the germline axis (x-axis) for all indicated genotypes. Error bars represent standard error of the mean.
(0.57 MB TIF)

Figure S7 High magnification images of RAD-51 foci on mid-pachytene nuclei. Mid-pachytene nuclei (zone 5) from whole mounted gonads of the indicated genotypes. DAPI-stained chromosomes (blue), α-RAD-51 (red). Bar, 5 µm.
(10.19 MB TIF)

Figure S8 Increased levels of RAD-51 foci and larger foci are observed in *him-18* mutants. Immunostaining of RAD-51 (red) on DAPI-stained chromosomes (blue) in nuclei at the premeiotic tip (zone 1) for the indicated genotypes. Bar, 5 µm.
(9.90 MB TIF)

Figure S9 *cep-1(RNAi)* suppresses the increased germ cell apoptosis observed in both *him-18* and *spo-11;him-18* mutants. (A) Quantitation of germline apoptosis by acridine orange staining. (B) Quantitation of germline apoptosis visualized in CED-1::GFP transgenic animals. *P*-values were assessed by the two tailed Mann-Whitney test; 95% C.I. n = number of gonad arms scored for each genotype.
(0.49 MB TIF)

Figure S10 *him-18* mutants do not display single strand breaks and show a normal checkpoint response. (A) Staining with anti-RPA-1 antibody (green) and DAPI (blue) in wild type and *him-18* mutants, and in wild type after treatment with 40 mM hydroxyurea as a positive control. Images are of the mitotic zone, transition zone and pachytene, from left to right. Bars, 5 µm. (B) Images showing the mitotic zone stained by DAPI in wild type and *him-18* mutants with no treatment and 16 hours after 24 hour treatment with 40 mM hydroxyurea. Bars, 15 µm. (C) Relative hatching of wild type and *him-18* mutants after treatment with the indicated doses of hydroxyurea (HU). Hatching is plotted as a fraction of the hatching observed in untreated animals. Error bars indicate standard error of the mean for 20 animals in each of two independent experiments.
(5.67 MB TIF)

Figure S11 Evidence of chromatin bridges with RAD-51 foci in *him-18;him-6* double mutants. (A) High magnification image of a chromatin bridge (indicated by the yellow arrow on the DAPI only panel) with RAD-51 foci observed in *him-18;him-6* double mutants at the premeiotic tip (zone 1). Bar, 5 µm. (B) Graph depicting the correlation coefficient (r) between the length of the chromatin bridges and the number of RAD-51 foci observed in *him-18;him-6*, *him-18;spo-11* and *xpf-1;him-18* mutants. All chromatin bridges observed in this study were plotted. r = 0.6. (C) Graph depicting the frequency of chromatin bridges observed per the total number of nuclei scored (y-axis) in each zone along the germline axis (x-axis) for the indicated genotypes.
(2.05 MB TIF)

Figure S12 HIM-18 interacts with SLX-1 and XPF-1 in several yeast two-hybrid conditions. (A) Y8800/Y8930 containing pDEST22-AD/pDEST32-DB. (B) AH109/Y189 containing pVV213-AD/pVV212-DB. (C) Mav203/Mav103 containing pVV213-AD/pVV212-DB. (D) Matrix indicates the pair-wise combinations for AD-X and DB-Y interactions examined at each position on the plates.
(4.68 MB TIF)

Figure S13 Loss of *him-18* does not enhance G/C tract deletion in the *dog-1* background. (A) Schematic representation of the G/C tract on the *vab-1* locus where the relative positions of the PCR primers are indicated. (B) Each lane represents the product of a PCR reaction performed on a single adult worm. Wild type, *him-18*, *dog-1*, and *dog-1; him-18* are shown. The asterisks indicate where deletion bands are present. Lanes labeled as M correspond to the DNA size marker.
(8.06 MB TIF)

Figure S14 Chromosome bridges and lagging chromosomes are observed at anaphase I in *him-18* mutants. High magnification images of DAPI-stained chromosomes at anaphase I in wild type and *him-18* mutants. Yellow arrows indicate chromosome bridges. To facilitate the visualization of these chromosomes bridges the same image was captured at a higher exposure as depicted in the smaller panel and indicated by arrowheads. n-values are indicated for each genotype. Bar, 2 µm.
(2.09 MB TIF)

Table S1 Diameter of germline mitotic nuclei in WT and *him-18* mutants
(0.02 MB DOC)

Table S2 *P*-values from the Fisher's Exact Test performed comparing crossover frequencies depicted in Figure 7A between wild type and either *him-18* or *xpf-1* mutants.
(0.02 MB DOC)
