# Supplementary figures and images for: Caenorhabditis elegans HIM-18/SLX-4 Interacts with SLX-1 and XPF-1 and Maintains Genomic Integrity in the Germline by Processing Recombination Intermediates
Source: PLoS Genet. 2009 Nov 20;5(11):e1000735. doi: 10.1371/journal.pgen.1000735 (PMC2770170; doi:10.1371/journal.pgen.1000735)

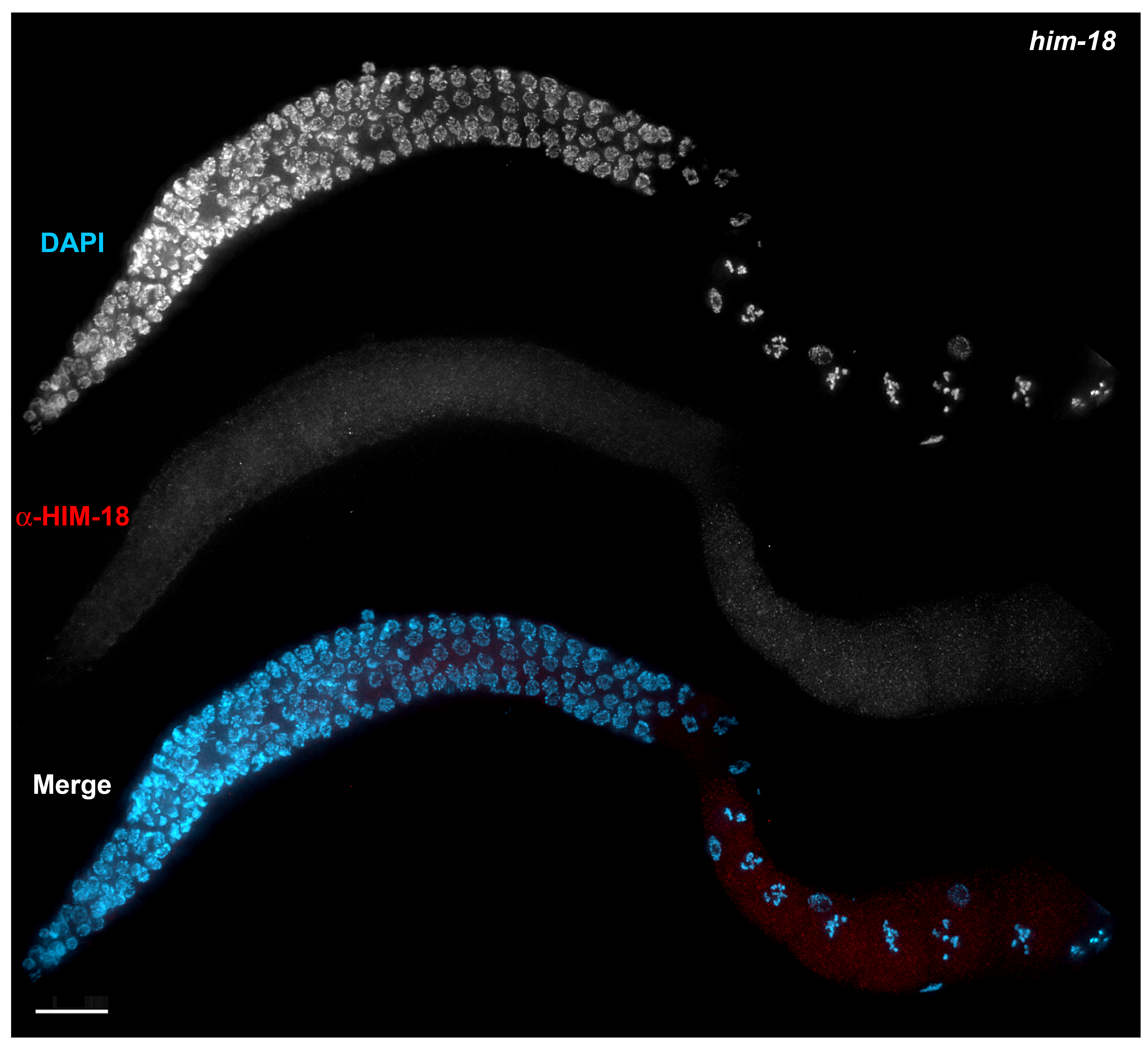

Supplement: Figure S1 — Immunostaining indicates lack of HIM-18 signal on the germline of him-18 mutants. Low magnification image of a whole mounted him-18 gonad where DAPI-stained chromosomes (blue) were immunostained with α-HIM-18 (red). Bar, 20 µm. (7.73 MB TIF) [file pgen.1000735.s001.tif]

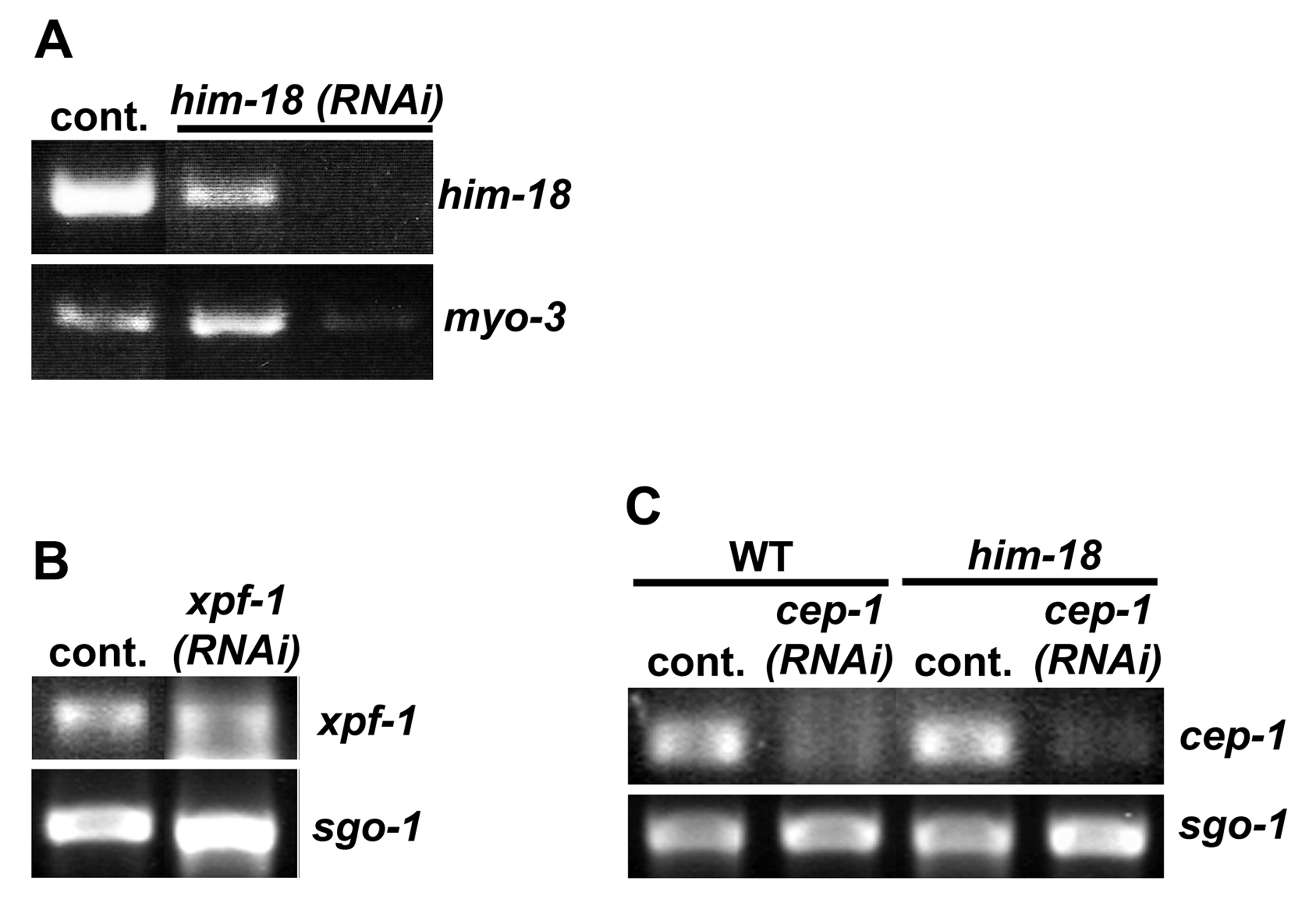

Supplement: Figure S2 — RT-PCR analysis of RNAi experiments. (A) RT-PCR in him-18(RNAi) worms compared to control (RNAi) worms. Feeding vector (pL4440) alone is the control (RNAi) indicated by ‘cont.’. myo-3 expression was used as a loading control. (B) RT-PCR in xpf-1(RNAi) worms. sgo-1 expression was used as a loading control. (C) RT-PCR in cep-1(RNAi) and cep-1(RNAi);him-18 worms. sgo-1 expression was used as a loading control. Each lane contains the RT-PCR products from single adult worms 22–24 hours post-L4. (1.14 MB TIF) [file pgen.1000735.s002.tif]

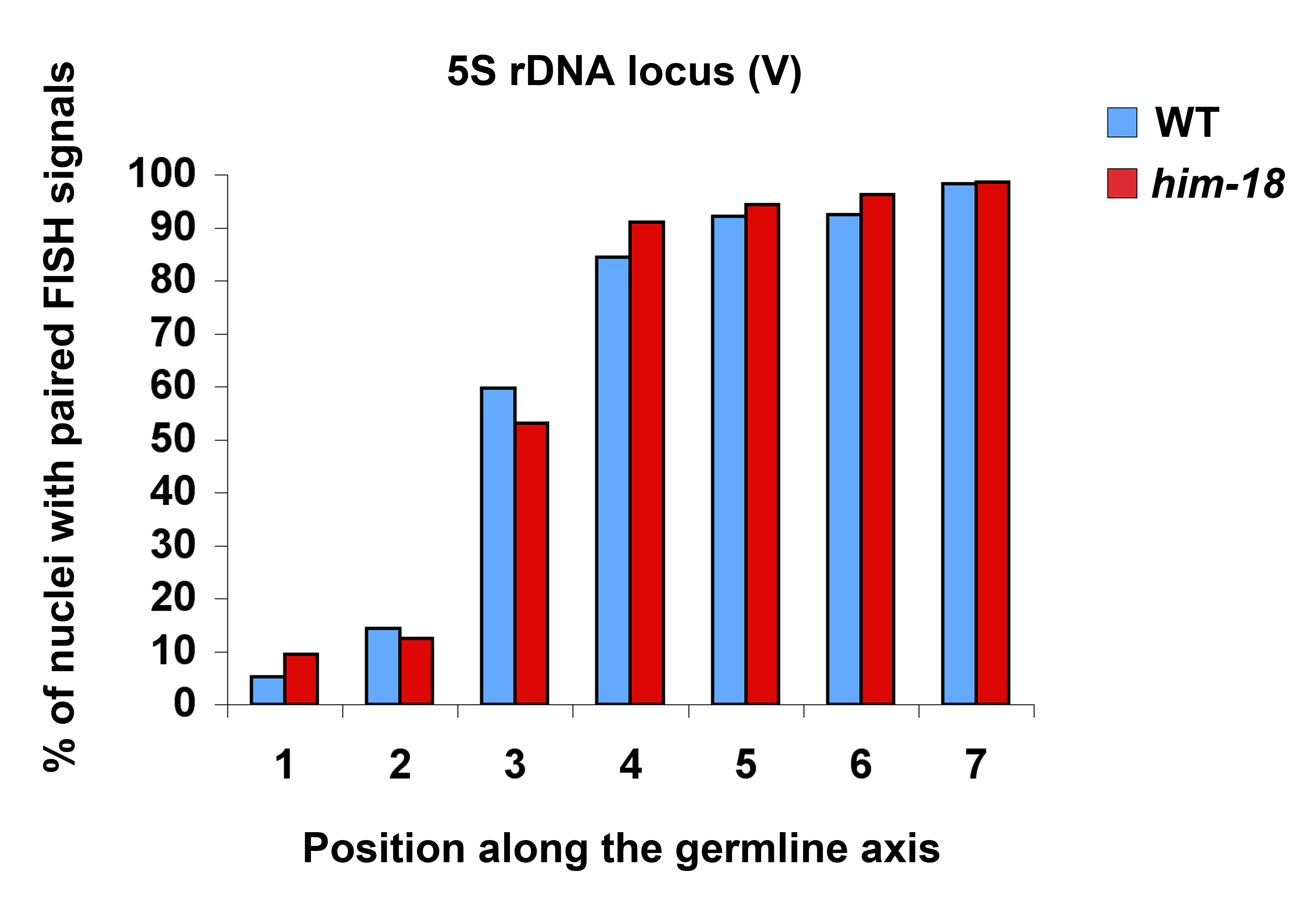

Supplement: Figure S3 — Quantitative analysis of homologous pairing in him-18 mutants. Quantitation of homologous chromosome pairing at the 5S rDNA locus on chromosome V. Graph depicts the percentage of nuclei carrying paired homologous chromosomes (y-axis) within each zone along the germline (x-axis). Homolog pairing levels are indistinguishable between wild type and him-18 mutants throughout meiotic prophase. Data was collected from wild type (n = 3) and him-18 (n = 4) whole mounted germlines examined by FISH. The average numbers of nuclei scored per zone for each genotype are as follows: zone 1, n = 101; zone 2, n = 135; zone 3, n = 125; zone 4, n = 113; zone 5, n = 104; zone 6, n = 81; zone 7, n = 65. (0.34 MB TIF) [file pgen.1000735.s003.tif]

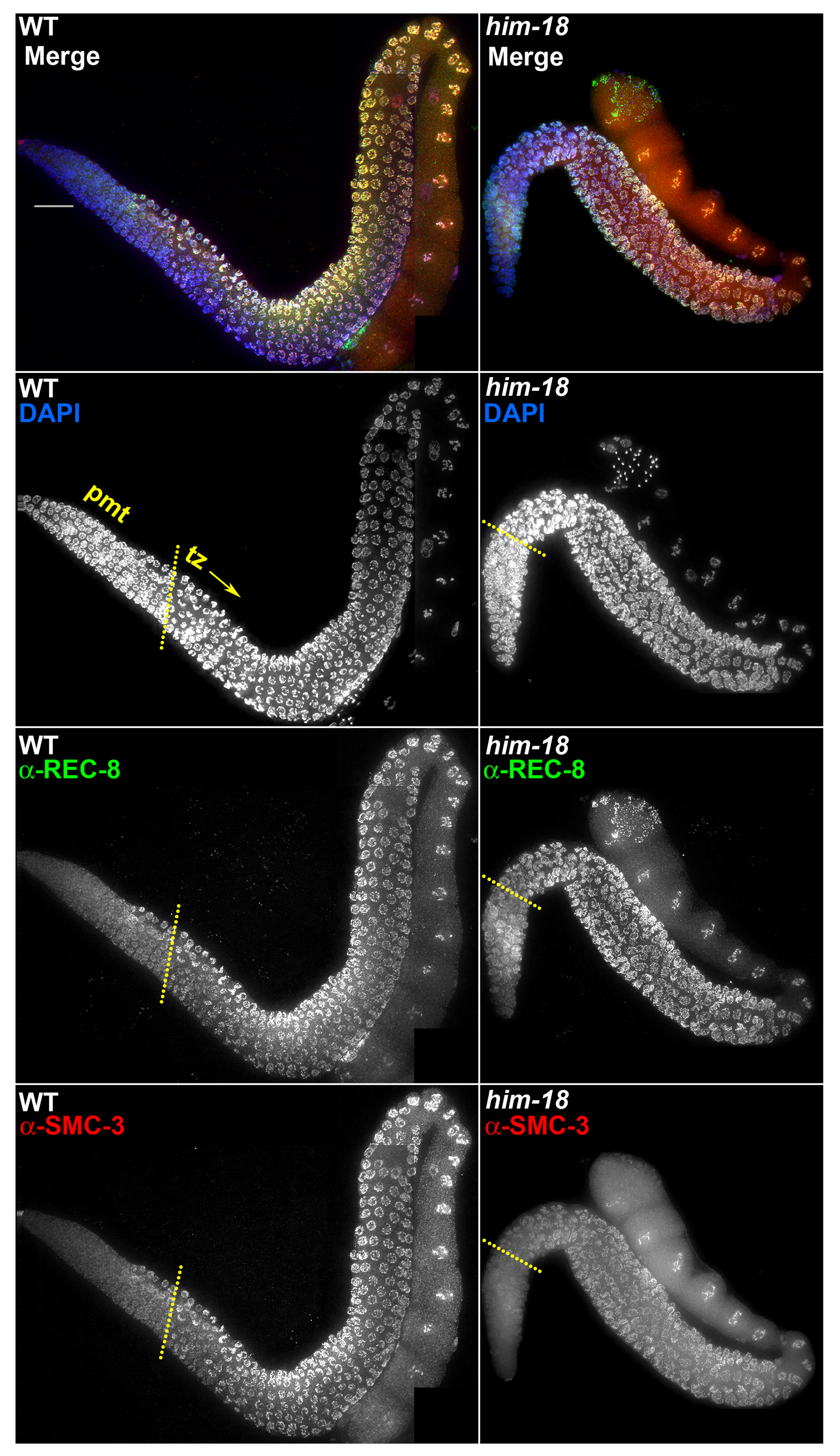

Supplement: Figure S4 — Axis morphogenesis is normal in him-18 mutants. Low magnification image of whole mounted wild type and him-18 gonads. DAPI-stained chromosomes (blue) were immunostained with α-REC-8 (green) and α-SMC-3 (red). The yellow dotted lines indicate the borders between the premeiotic tip (pmt) and the transition zone (tz). The direction of meiotic progression is indicated by the yellow arrow. Bar, 20 µm. (9.99 MB TIF) [file pgen.1000735.s004.tif]

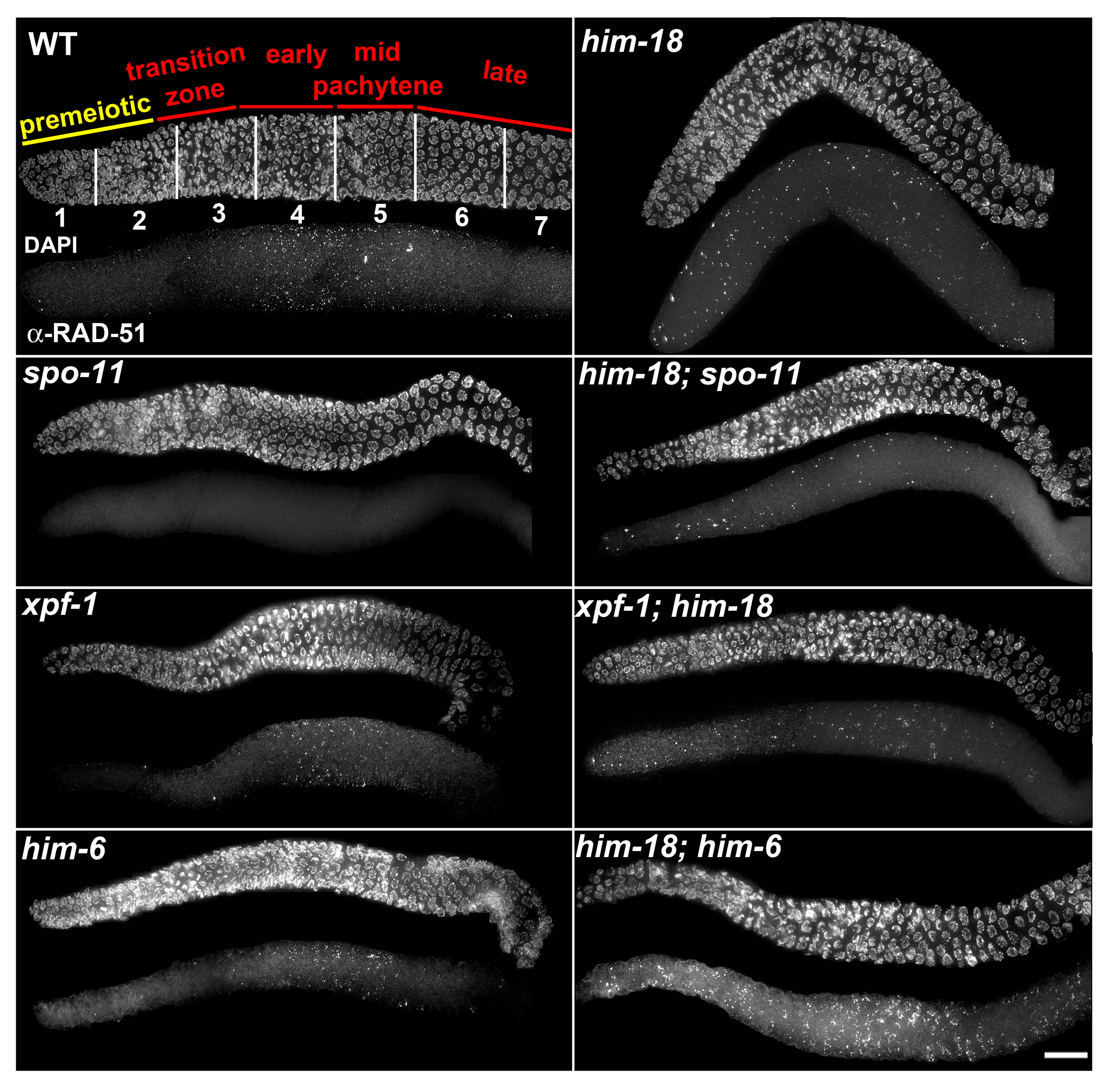

Supplement: Figure S5 — HIM-18 is required for DNA repair in both mitotic and meiotic germ cells. Low magnification images of germlines of the indicated genotypes immunostained with RAD-51 and DNA counterstained with DAPI. The seven zones in which RAD-51 foci are quantitated are depicted on the wild type germline. Bar, 20 µm. (9.14 MB TIF) [file pgen.1000735.s005.tif]

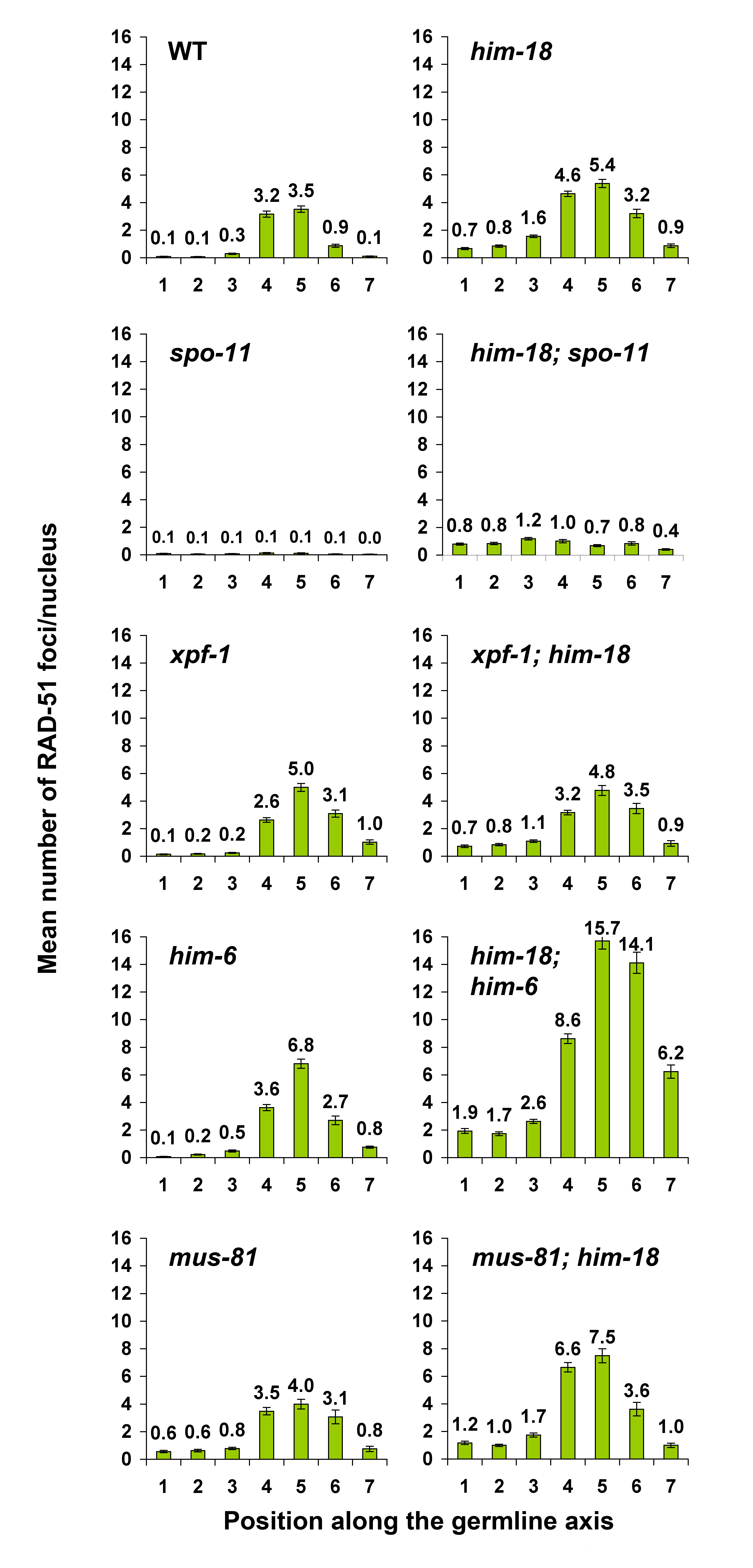

Supplement: Figure S6 — Mean number of RAD-51 foci per nucleus. Quantitative analysis of RAD-51 foci depicted in Figure 3, is represented here as the mean number of RAD-51 foci observed per nucleus (y-axis) on each zone along the germline axis (x-axis) for all indicated genotypes. Error bars represent standard error of the mean. (0.57 MB TIF) [file pgen.1000735.s006.tif]

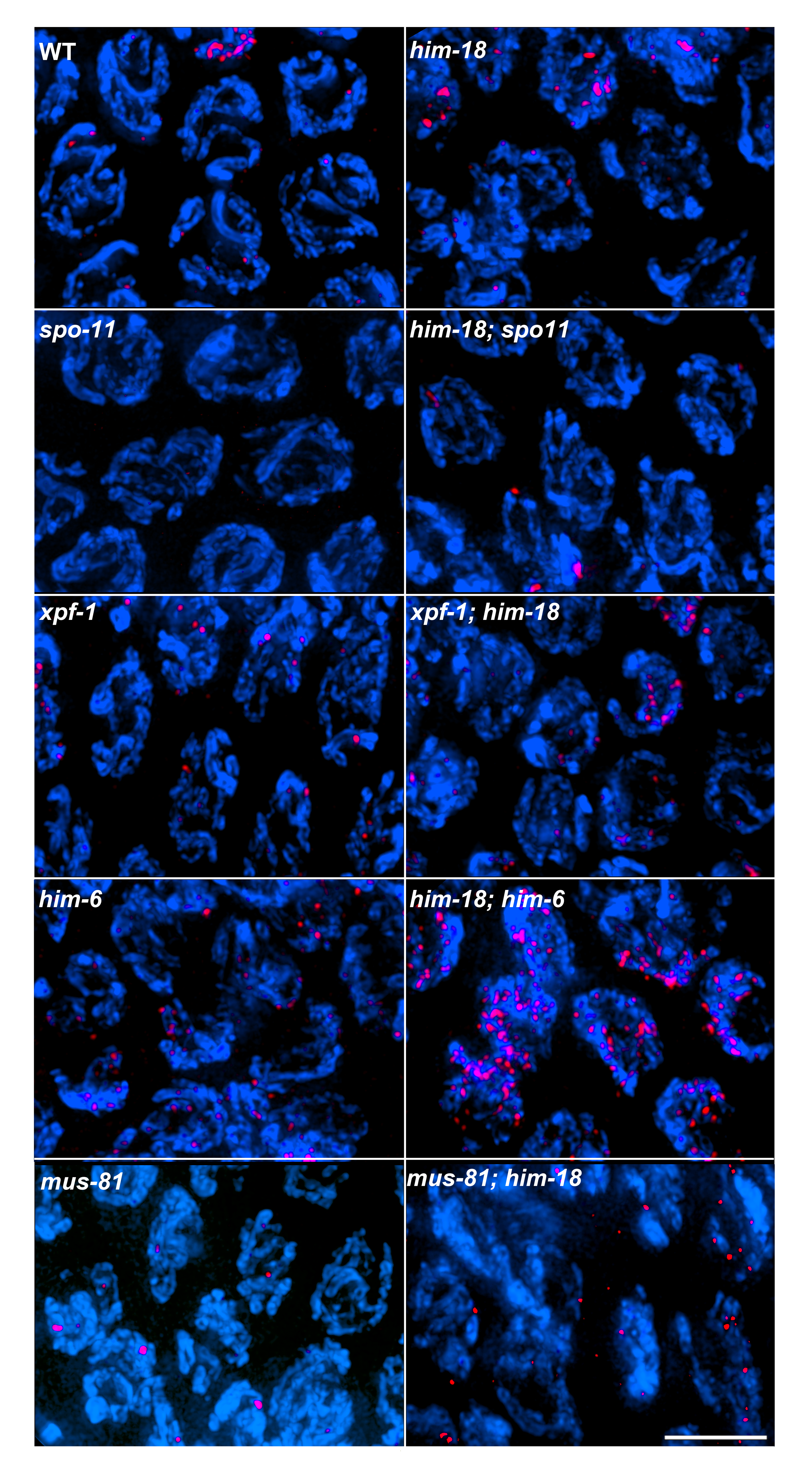

Supplement: Figure S7 — High magnification images of RAD-51 foci on mid-pachytene nuclei. Mid-pachytene nuclei (zone 5) from whole mounted gonads of the indicated genotypes. DAPI-stained chromosomes (blue), α-RAD-51 (red). Bar, 5 µm. (10.19 MB TIF) [file pgen.1000735.s007.tif]

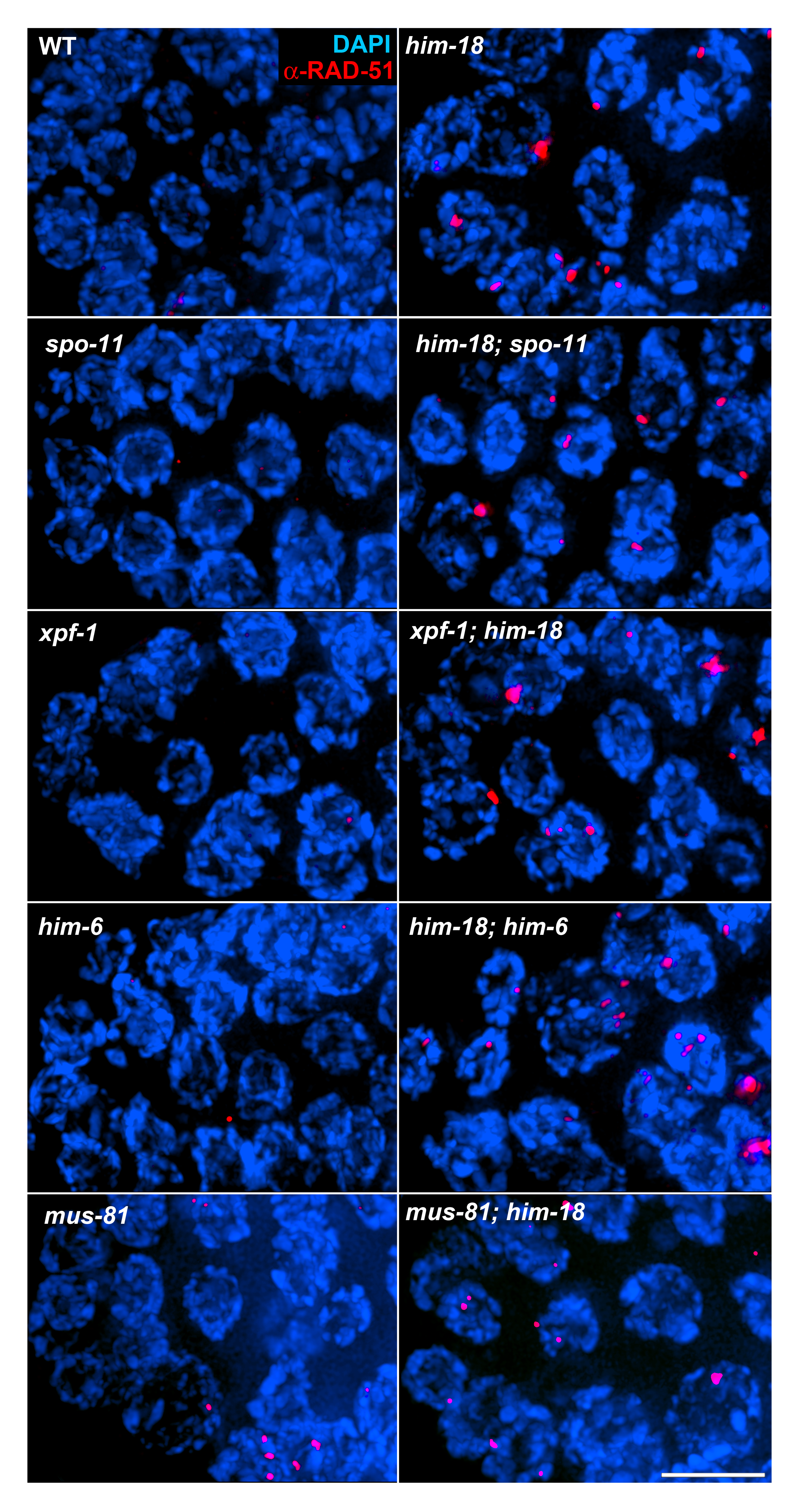

Supplement: Figure S8 — Increased levels of RAD-51 foci and larger foci are observed in him-18 mutants. Immunostaining of RAD-51 (red) on DAPI-stained chromosomes (blue) in nuclei at the premeiotic tip (zone 1) for the indicated genotypes. Bar, 5 µm. (9.90 MB TIF) [file pgen.1000735.s008.tif]

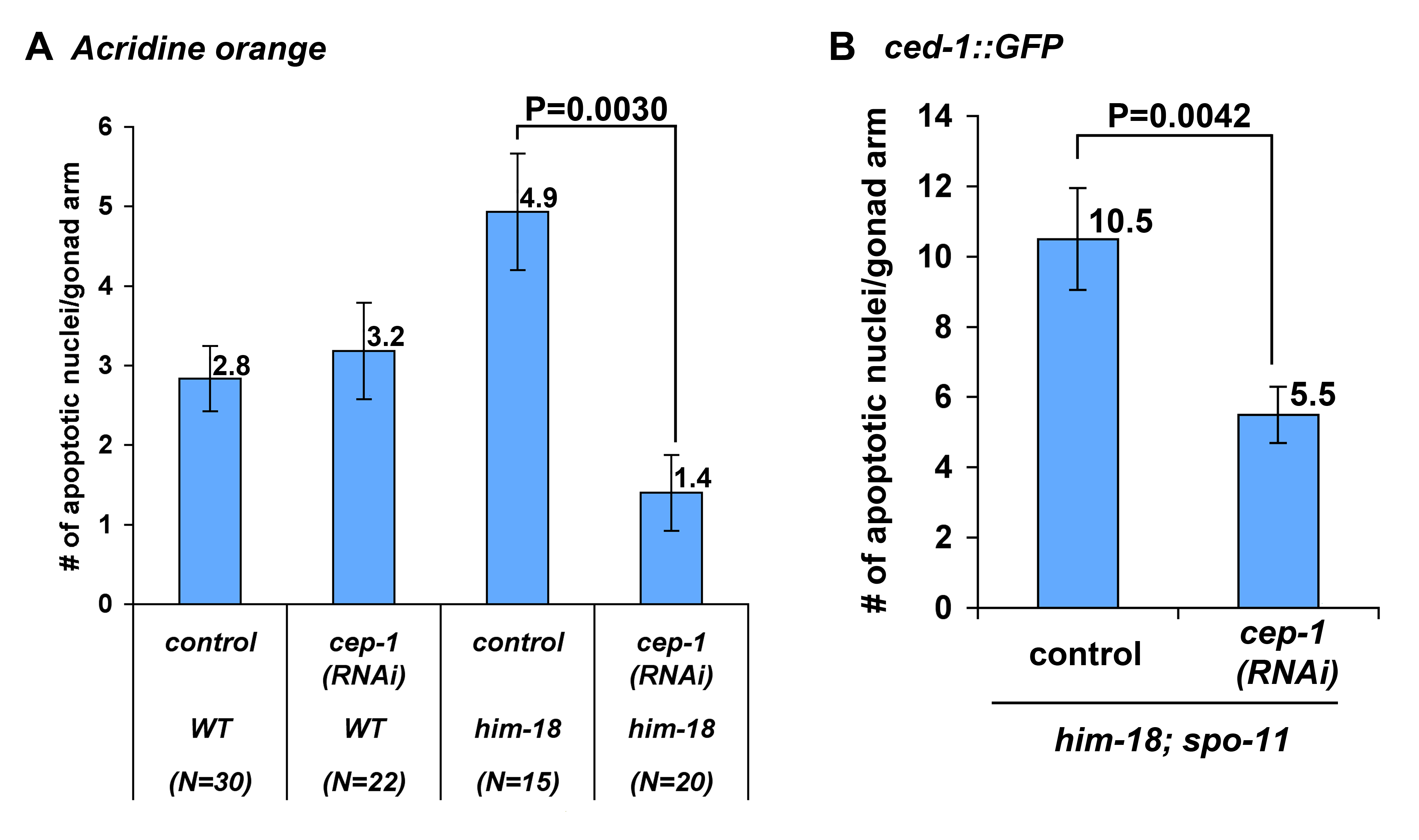

Supplement: Figure S9 — cep-1(RNAi) suppresses the increased germ cell apoptosis observed in both him-18 and spo-11;him-18 mutants. (A) Quantitation of germline apoptosis by acridine orange staining. (B) Quantitation of germline apoptosis visualized in CED-1::GFP transgenic animals. P-values were assessed by the two tailed Mann-Whitney test; 95% C.I. n = number of gonad arms scored for each genotype. (0.49 MB TIF) [file pgen.1000735.s009.tif]

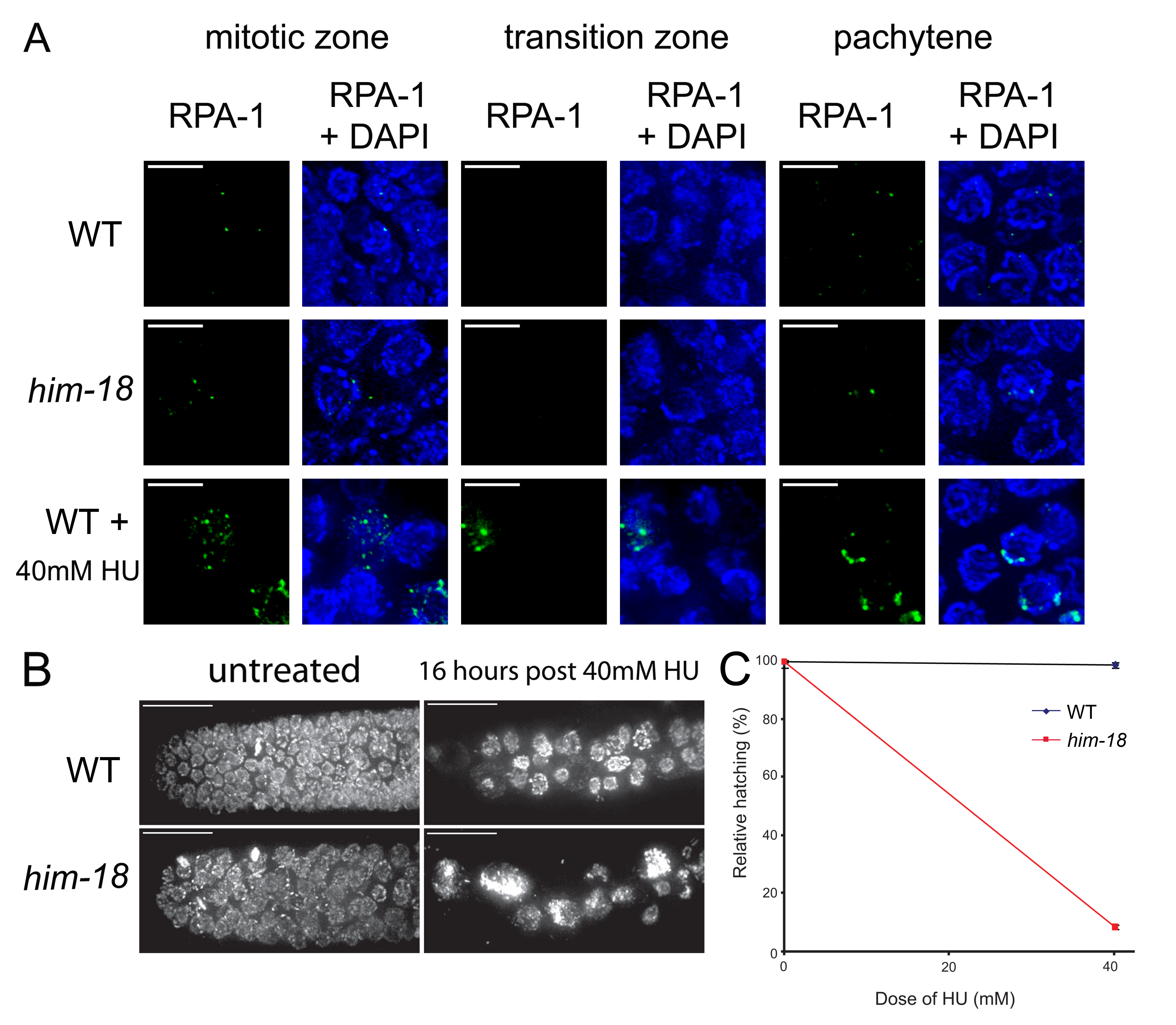

Supplement: Figure S10 — him-18 mutants do not display single strand breaks and show a normal checkpoint response. (A) Staining with anti-RPA-1 antibody (green) and DAPI (blue) in wild type and him-18 mutants, and in wild type after treatment with 40 mM hydroxyurea as a positive control. Images are of the mitotic zone, transition zone and pachytene, from left to right. Bars, 5 µm. (B) Images showing the mitotic zone stained by DAPI in wild type and him-18 mutants with no treatment and 16 hours after 24 hour treatment with 40 mM hydroxyurea. Bars, 15 µm. (C) Relative hatching of wild type and him-18 mutants after treatment with the indicated doses of hydroxyurea (HU). Hatching is plotted as a fraction of the hatching observed in untreated animals. Error bars indicate standard error of the mean for 20 animals in each of two independent experiments. (5.67 MB TIF) [file pgen.1000735.s010.tif]

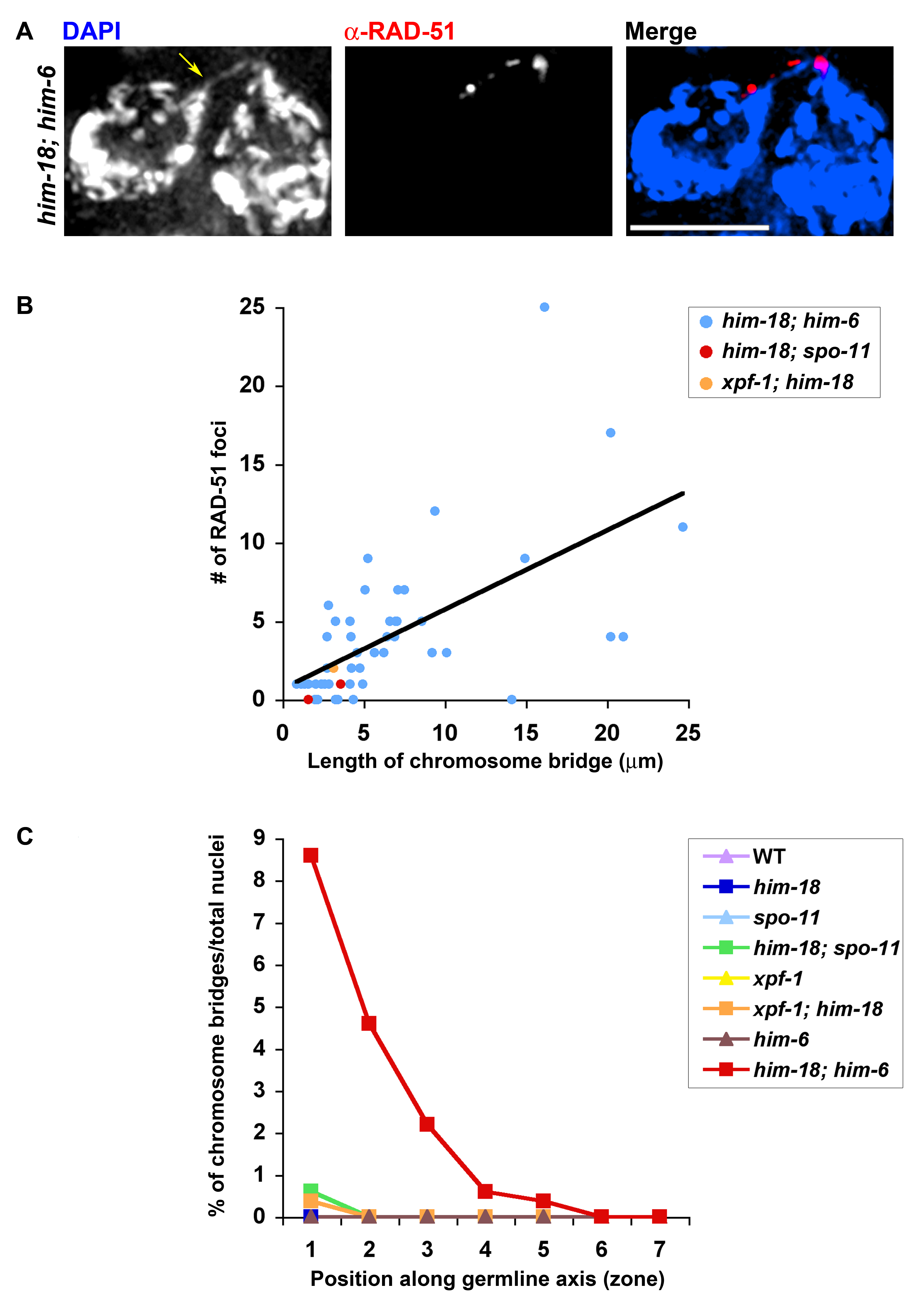

Supplement: Figure S11 — Evidence of chromatin bridges with RAD-51 foci in him-18;him-6 double mutants. (A) High magnification image of a chromatin bridge (indicated by the yellow arrow on the DAPI only panel) with RAD-51 foci observed in him-18;him-6 double mutants at the premeiotic tip (zone 1). Bar, 5 µm. (B) Graph depicting the correlation coefficient (r) between the length of the chromatin bridges and the number of RAD-51 foci observed in him-18;him-6, him-18;spo-11 and xpf-1;him-18 mutants. All chromatin bridges observed in this study were plotted. r = 0.6. (C) Graph depicting the frequency of chromatin bridges observed per the total number of nuclei scored (y-axis) in each zone along the germline axis (x-axis) for the indicated genotypes. (2.05 MB TIF) [file pgen.1000735.s011.tif]

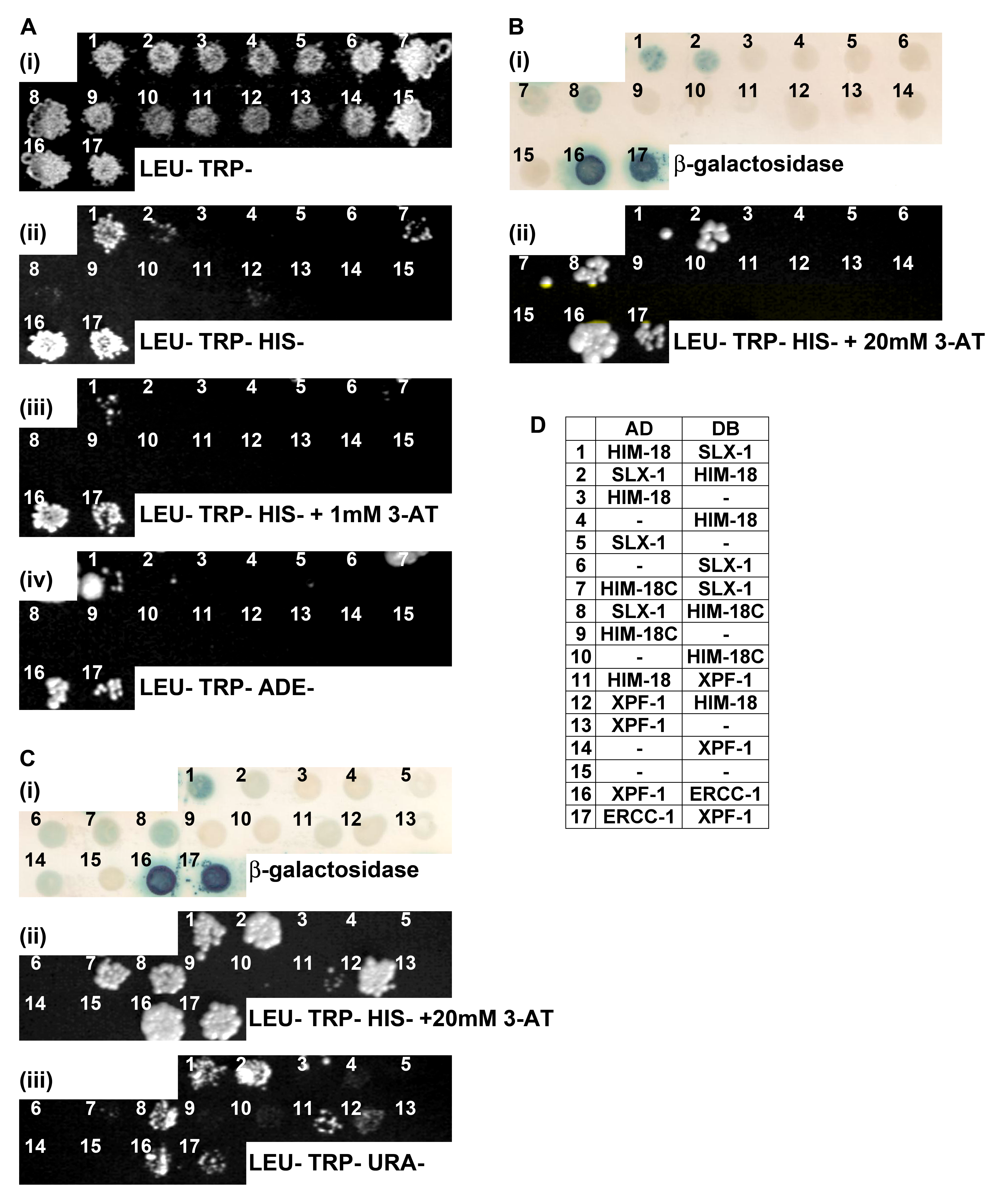

Supplement: Figure S12 — HIM-18 interacts with SLX-1 and XPF-1 in several yeast two-hybrid conditions. (A) Y8800/Y8930 containing pDEST22-AD/pDEST32-DB. (B) AH109/Y189 containing pVV213-AD/pVV212-DB. (C) Mav203/Mav103 containing pVV213-AD/pVV212-DB. (D) Matrix indicates the pair-wise combinations for AD-X and DB-Y interactions examined at each position on the plates. (4.68 MB TIF) [file pgen.1000735.s012.tif]

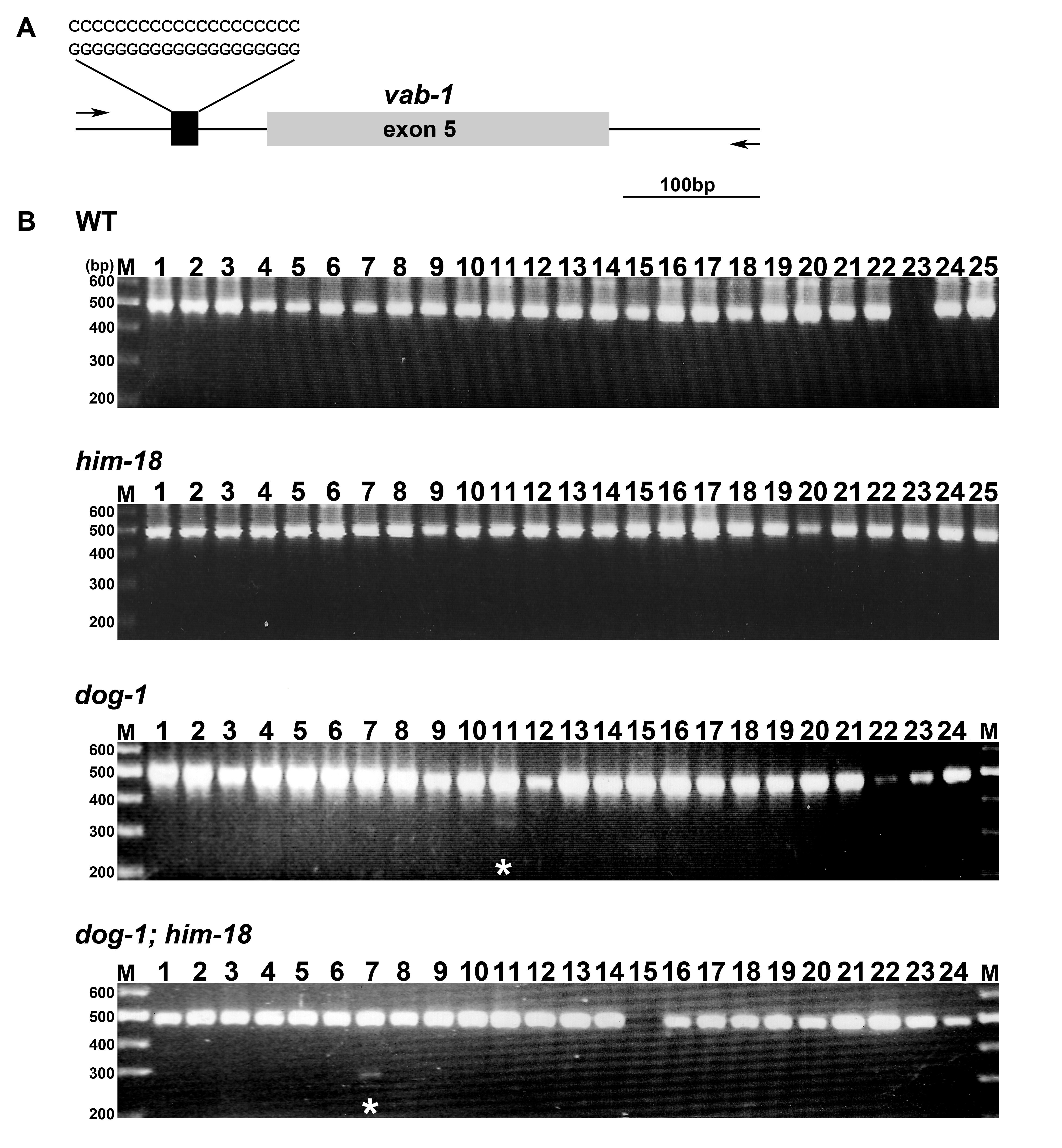

Supplement: Figure S13 — Loss of him-18 does not enhance G/C tract deletion in the dog-1 background. (A) Schematic representation of the G/C tract on the vab-1 locus where the relative positions of the PCR primers are indicated. (B) Each lane represents the product of a PCR reaction performed on a single adult worm. Wild type, him-18, dog-1, and dog-1; him-18 are shown. The asterisks indicate where deletion bands are present. Lanes labeled as M correspond to the DNA size marker. (8.06 MB TIF) [file pgen.1000735.s013.tif]

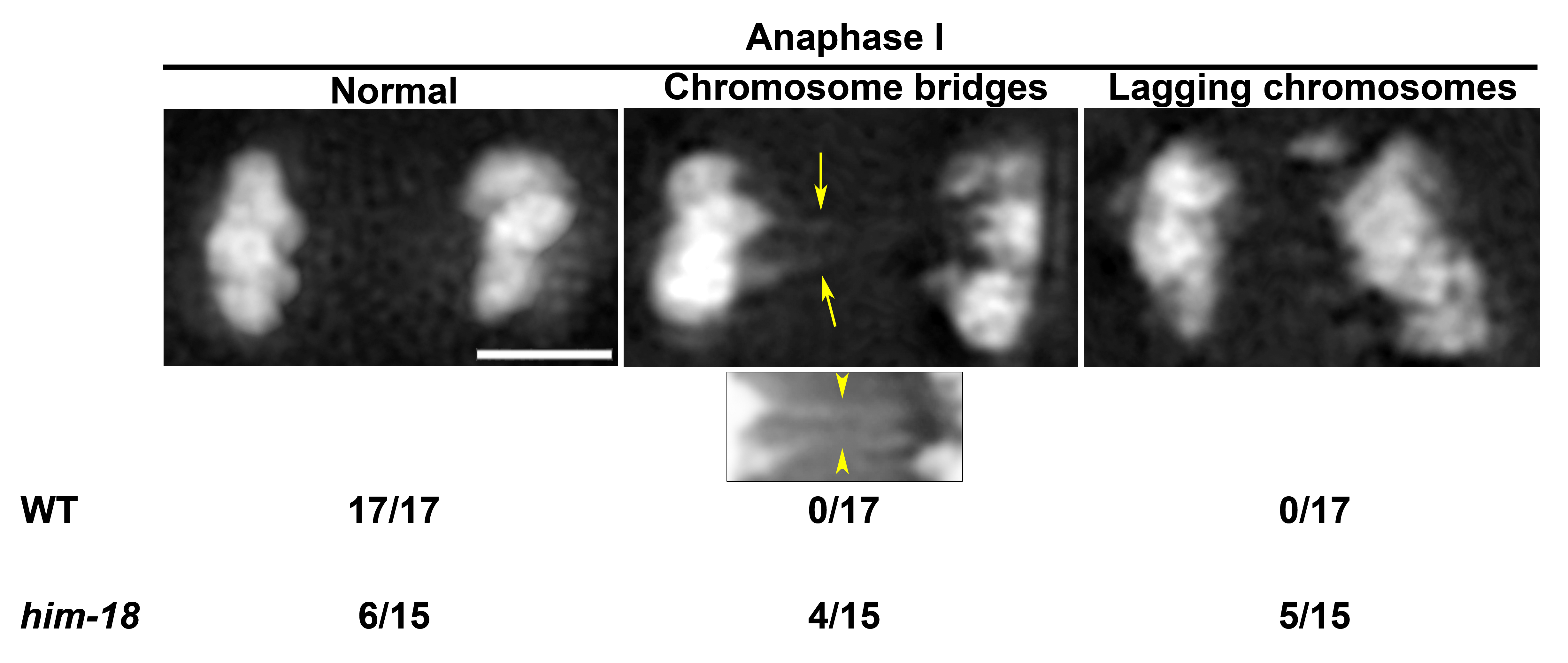

Supplement: Figure S14 — Chromosome bridges and lagging chromosomes are observed at anaphase I in him-18 mutants. High magnification images of DAPI-stained chromosomes at anaphase I in wild type and him-18 mutants. Yellow arrows indicate chromosome bridges. To facilitate the visualization of these chromosomes bridges the same image was captured at a higher exposure as depicted in the smaller panel and indicated by arrowheads. n-values are indicated for each genotype. Bar, 2 µm. (2.09 MB TIF) [file pgen.1000735.s014.tif]
