# Supplementary material for: Caenorhabditis elegans HIM-18/SLX-4 Interacts with SLX-1 and XPF-1 and Maintains Genomic Integrity in the Germline by Processing Recombination Intermediates
Source: PLoS Genet. 2009 Nov 20;5(11):e1000735. doi: 10.1371/journal.pgen.1000735 (PMC2770170; doi:10.1371/journal.pgen.1000735)
Supplement: Table S1 — Diameter of germline mitotic nuclei in WT and him-18 mutants (0.02 MB DOC) [file pgen.1000735.s015.doc]

**Table S1. Diameter of germline mitotic nuclei in WT and *him-18* mutants**

| **Genotype** | **Average diameter (μm) ^a^** | **Average largest nuclear diameter (μm) ^b^** |
| --- | --- | --- |
| WT (n=222) | 3.17 ± 0.04 | 4.40 ± 0.17 |
| *him-18* (n=256) | 3.91 ± 0.05 | 5.46 ± 0.22 |

5 and 10 gonads (zone 1) were analyzed for WT and *him-18* mutants, respectively. All values are ± standard error of the mean. ^a^ *P* < 0.0001,  ^b^ *P* = 0.0047 (by the two tailed Mann-Whitney test; 95% C.I.).
