# Supplementary material for: Caenorhabditis elegans HIM-18/SLX-4 Interacts with SLX-1 and XPF-1 and Maintains Genomic Integrity in the Germline by Processing Recombination Intermediates
Source: PLoS Genet. 2009 Nov 20;5(11):e1000735. doi: 10.1371/journal.pgen.1000735 (PMC2770170; doi:10.1371/journal.pgen.1000735)
Supplement: Table S2 — P-values from the Fisher's Exact Test performed comparing crossover frequencies depicted in Figure 7A between wild type and either him-18 or xpf-1 mutants. (0.02 MB DOC) [file pgen.1000735.s016.doc]

**Table S2.** *P*-values from the Fisher's Exact Test performed comparing crossover frequencies depicted in Figure 7A between wild type and either *him-18* or *xpf-1* mutants.

| **Ch. I** | **A-B** | **B-C** | **C-D** | **D-E** | **A-E** |
| --- | --- | --- | --- | --- | --- |
| *him-18* | 0.1471 | 0.4018 | **0.0341** | 0.3187 | **0.0005** |
| *xpf-1* | 0.0747 | 0.4231 | 0.2946 | 0.1347 | **0.0302** |

| **Ch. X** | **A-B** | **B-C** | **C-D** | **D-E** | **A-E** |
| --- | --- | --- | --- | --- | --- |
| *him-18* | 0.1166 | 0.1319 | 0.2160 | **0.0002** | **<0.0001** |
| *xpf-1* | 0.1994 | 0.6278 | 1.0000 | 0.1701 | **0.0434** |

Intervals are as indicated in Figure 7A. The significance of the crossover frequencies was assessed by the Fisher's Exact Test (two-sided *P* value and 95% confidence intervals) using the InStat software (Graphpad).
